# Denosumab as an immune modulator in HER2-negative early breast cancer: results of the window-of-opportunity D-BIOMARK clinical trial

**DOI:** 10.1186/s13058-025-01996-w

**Published:** 2025-05-12

**Authors:** Andrea Vethencourt, Eva M. Trinidad, Eduard Dorca, Anna Petit, M. Teresa Soler-Monsó, Marina Ciscar, Alexandra Barranco, Gema Pérez-Chacón, María Jimenez, Mario Rodríguez, Clara Gomez-Aleza, Elvira Purqueras, Enrique Hernández-Jiménez, Ander Urruticoechea, Idoia Morilla, Isaac Subirana, Amparo García-Tejedor, Miguel Gil-Gil, Sonia Pernas, Catalina Falo, Eva Gonzalez-Suarez

**Affiliations:** 1 Breast Cancer Unit, Medical Oncology Department, Institut Català d’Oncologia, Barcelona, Spain; 2 IDIBELL, Institut d’Investigació Biomèdica de Bellvitge, Barcelona, Spain; 3 Faculty of Medicine and Health Sciences, Universitat de Barcelona, Barcelona, Spain; 4 Pathology Department and Breast Cancer Unit, Hospital Universitari de Bellvitge and Institut Català d’Oncologia, Barcelona, Spain; 5 Centro Nacional de Investigaciones Oncológicas (CNIO), Madrid, Spain; 6 Gipuzkoa Cancer Unit, OSID-Onkologikoa, Osakidetza, Donostia, Spain; 7 Medical Oncology Department, Hospital Universitario de Navarra, Navarra, Spain; 8 Statistician Department, Researchmar, Barcelona, Spain; 9 Gynecology Department and Breast Cancer Unit, Hospital Universitari de Bellvitge and Institut Català d’Oncologia, Barcelona, Spain

**Keywords:** Breast cancer, RANK, RANKL, Denosumab, TILs, Immune enhancer, Tumor cell proliferation, Tumor cell survival, HER2-negative

## Abstract

**Background:**

The RANK pathway has been extensively investigated for its role in bone resorption; however, its significance extends beyond bone metabolism. Preclinical models suggest that inhibition of RANK signaling can prevent mammary tumor development by reducing proliferation and tumor cell survival. Additionally, both preclinical and clinical data support the ability of RANK pathway inhibitors to enhance the anti-tumor immune response.

**Methods:**

D-BIOMARK is a prospective, randomized window-of-opportunity clinical trial assessing the biological effects of denosumab, a monoclonal antibody against RANKL, in patients with HER2-negative early breast cancer. The study aims to assess denosumab’s impact on breast tumor cell proliferation, apoptosis, and its potential to influence the tumor immune microenvironment. A total of 60 patients were enrolled and randomized 2:1 to receive two doses of single agent denosumab (120 mg one week apart) before surgery or to the control arm (no treatment). Fifty-eight patients were evaluated, 27 pre-menopausal and 31 post-menopausal women, 48 with luminal tumors and 10 with triple negative breast cancer. Paired tumor samples were collected to compare baseline (core biopsy) and surgical (surgical specimen) time points, as well as serum samples at both time points.

**Results:**

Denosumab demonstrated its ability to reduce serum free RANKL levels (experimental *p* < 0.001, control *p* = 0.270). However, a reduction in tumor cell proliferation or cell survival was not observed. A denosumab-driven increase in tumor infiltrating lymphocytes (TILs) was observed (experimental *p* = 0.001, control *p* = 0.060), particularly in the luminal B-like population (experimental *p* = 0.012, control *p* = 0.070) and a similar trend in the TNBC group (experimental *p* = 0.079, control *p* = 0.237). Denosumab led to increased TILs in both pre-menopausal (experimental *p* = 0.048, control *p* = 0.639) and post-menopausal (experimental *p* = 0.041, control *p* = 0.062) women with luminal tumors. RANK protein expression in tumor and stroma was associated with markers of tumor aggressiveness but an increase in TILs was observed in the experimental arm, irrespectively of RANK and RANKL expression in tumor or stromal cells.

**Conclusions:**

The D-BIOMARK trial suggests a potential role for denosumab as an immune-enhancing agent in early HER2-negative breast cancer. Although preoperative denosumab did not reduce tumor proliferation or increased apoptosis, it led to an increase in TILs, particularly in luminal B-like tumors. These findings underscore the importance of further investigation into the multifaceted aspects of the RANK pathway.

*Trial registration* EudraCT number: 2016-002678-11 registered on June 15, 2018. ClinicalTrials.gov identifier: NCT03691311, retrospectively registered on September 04, 2018.

**Supplementary Information:**

The online version contains supplementary material available at 10.1186/s13058-025-01996-w.

## Introduction

The receptor activator of nuclear factor κB, known as RANK, member of tumor necrosis factor receptor (TNFR) superfamily, and its ligand (RANKL) have emerged as potential therapeutic targets in breast cancer (BC) and other solid tumors [1–3].

Binding of RANKL to RANK leads to activation of signaling pathways related to tumor proliferation, survival, and inflammation, such as the canonical and non-canonical NFκB, MAPK and PI3K-AKT. Osteoprotegerin (OPG), a natural negative regulator of the RANK pathway acts as a decoy receptor and prevents the binding of RANKL to RANK. The RANK/RANKL/OPG axis has been widely studied as a regulator of bone resorption, leading to the development of denosumab, a highly specific immunoglobulin type IgG2 monoclonal antibody, which binds with high affinity to human RANKL and neutralizes its activity [4]. Denosumab is approved for the prevention of skeletal events in patients with bone metastases and for the treatment of unresectable giant cell tumors of bone and osteoporosis [3, 5, 6].

Preclinical data show that RANK signaling regulates mammary gland development and mammary cell fate [7–10]. RANKL is the main mediator of the proliferative and pro-tumorigenic role of progesterone in the mammary gland. Pharmacological or genetic inhibition of the pathway prevents or attenuates mammary tumor appearance, reduces cell proliferation in preneoplastic lesions and tumor cell survival in mouse adenocarcinomas [2, 11]. Blockade of the RANK pathway also reduces the incidence of lung metastases and enhances the differentiation of tumor cells in mouse models [2, 11–14].

In human mammary adenocarcinomas, RANK protein expression, detected by immunohistochemistry (IHC), is found in 15-20% of estrogen receptor (ER)-positive tumors and in 40% of ER-negative breast adenocarcinomas. RANK protein expression in tumor cells associates with an aggressive tumor phenotype, including hormone receptor-negative tumors, high histological grade and high proliferative index [15–19]. Recent findings underscore that RANK protein expression in tumor cells serves as an independent maker of adverse prognosis in post-menopausal patients and ER-negative BC [19].

RANK signaling plays a crucial role in regulating the delicate balance between tolerance and immunity [20]. RANK is predominantly expressed by myeloid cells such as dendritic cells and macrophages while RANKL expression has been observed on T cells in both the tumor microenvironment (TME) and locoregional lymph nodes.

Despite strong preclinical evidence, the therapeutic benefit of denosumab in BC patients beyond its bone-related effects is unclear. In the adjuvant setting, the phase III ABCSG-18 clinical trial demonstrated that administering denosumab at 60 mg every 6 months for 5 years, alongside an aromatase inhibitor, not only delayed bone fractures and improved mineral bone density but also increased 8-year disease-free survival (DFS) from 77.5% to 80.6%, with a hazard ratio (HR) of 0.82 (95% confidence interval CI=0.69-1.98), *p*=0.02. In 2022, further analysis confirmed these benefits, including progression-free survival, bone metastasis-free survival, and overall survival, leading to a recommendation for routine use of denosumab as adjuvant therapy in post-menopausal women with hormone receptor-positive BC [21–23]. In contrast, in the D-CARE clinical trial, with adjuvant denosumab at a dose of 120 mg every 4 weeks during 6 months and then every 12 weeks up to five years, did not show changes in bone metastasis-free survival (HR 0.97 (95% IC 0.82–1.14), *p*=0.70) and reported a similar 5-year DFS between the two groups (HR: 1.04 (95% IC 0.91–1.19), *p*=0.57) [24]. Both studies involved different populations and yielded discordant results. In the neoadjuvant context in the GeparX study, the addition of denosumab to neoadjuvant treatment did not increase the pathological complete response (pCR) rate in early BC [25–28].

The D-BEYOND, a prospective single-arm window-of-opportunity trial, evaluated the biological effect of denosumab in pre-menopausal women diagnosed with early BC. It included 27 patients, only one was triple negative breast cancer (TNBC). All patients received two subcutaneous injections of denosumab (120 mg/dose) separated by one week prior to breast surgery. The baseline biopsies were compared with the surgical samples. The study did not meet its primary aims: a reduction in cell proliferation nor an increase in cell apoptosis was observed. However, a brief course of denosumab induced an increase in the inflammatory infiltrate measured by tumor-infiltrating lymphocytes (TILs), especially CD8+ T lymphocytes [29].

Here, we present results from the D-BIOMARK trial (NCT03691311), a window-of-opportunity study designed to assess the biological activity of single-agent denosumab in patients with primary operable HER2-negative BC.

## Materials and methods

### Trial design and patients

D-BIOMARK (NCT03691311) is a prospective, single institution, randomized window-of-opportunity clinical trial evaluating the biological effects of single-agent denosumab in treatment-naive patients with early HER2-negative BC who were candidates for tumor excision as the first therapeutic approach. Exclusion criteria included osteonecrosis of the jaw or risk of developing it, other active malignancies, hypocalcemia, or known hypersensitivity to denosumab. Randomization was stratified by menopausal status and ER-status (ER+ vs. triple negative). Post-menopausal was defined clinically as more than 1 year with amenorrhea, or ≥ 60 years old [30]. Early-stage breast cancer is defined as stage I, stage IIA, stage IIB, and stage IIIA breast cancers, which denote cancer that has not spread beyond the breast or the axillary lymph nodes [31].

**Fig. 1:**
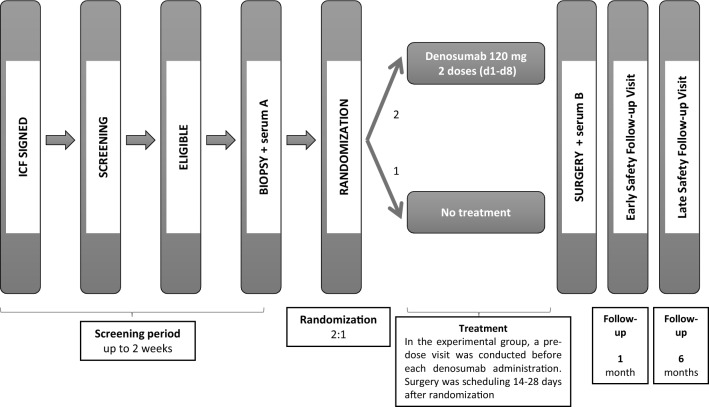
D-BIOMARK trial design. Diagram showing the steps to be followed by a patient from the time the Informed Consent Form (ICF) is signed until the end of the study

Written informed consent was obtained from all patients prior to any procedure within the study. Patients were randomized 2:1 to the experimental or control arm; the experimental group received two subcutaneous doses of denosumab (120 mg each) administered 7 days apart, while the control group received no treatment. We decided to administer the 120 mg dose, which is the standard dose approved for cancer patients. Despite the absence of prior data at the time of protocol drafting, a second dose was included to ensure sustained pharmacologically active blood levels, potentially serving as a loading dose. Screening visits were conducted for all participants. In both arms, a biopsy was performed at the time of diagnosis (referred to as “biopsy”), with serum collected also at baseline (referred to as “serum A”). Furthermore, a secondary biopsy of the surgical specimen was performed two to four weeks after enrollment, at the time of the surgical excision of the breast tumor (referred to as “surgery”), accompanied by a second blood sample collection (referred to as “serum B”). Additional visits and blood analyses were conducted in the experimental arm before each treatment session. Both groups underwent two follow-up visits for safety assessment at one month and six months post-surgery (Figure 1). Patients in the experimental group received daily calcium supplements (≥ 500 mg elemental calcium) and vitamin D (≥ 400 IU) for one month following the first dose to prevent hypocalcemia. The study protocol was approved by the institutional ethics committee (Research Ethics Committee of the Hospital Universitari de Bellvitge) protocol code PR035/21 and conducted in accordance with the ethical standards outlined in the 1964 Declaration of Helsinki.

Clinical data and biological characteristics of the tumors were prospectively obtained from medical records and pathology reports, respectively. Adverse events were recorded starting from the day of obtaining signed informed consent and continued until six months following surgery. The safety data were assessed in accordance with the National Cancer Institute Common Terminology Criteria for Adverse Events (NCI-CTCAE v5.0).

### Tumor assessment

The evaluation of conventional BC markers, including ER, progesterone receptor (PR), HER2, and Ki67, was performed in the Pathology Department of the Hospital Universitari de Bellvitge. The status of ER and PR was defined according to the guidelines of the American Society of Clinical Oncology and the College of American Pathologists (ASCO-CAP 2010) [32]. The histological grade was evaluated following Nottingham classification [33]. The surrogate subtypes of BC were defined according to St Gallen 2015 consensus meetings, using IHC substitutes as follows: luminal A-like: ER and/or PR(+), HER2(−), Ki67 < 20%; luminal B-like: ER and/or PR(+), HER2(−), Ki67 ≥ 20; TNBC: ER(−), PR(−), and HER2(−), regardless of the Ki67 score [34].

The objectives of this trial were tested by comparing the diagnostic biopsy (biopsy) with the surgical specimen (surgery). The evaluation of tumor cellularity was assessed in hematoxylin-eosin (H&E) staining tissue sections. For patients with multiple samples, the sample with the highest tumor content was chosen. The percentage of Ki67, cleaved caspase-3 and TILs were independently evaluated by at least two pathologists specialized in BC, blinded to clinical and experimental data. For Ki67, the antibody used was MIB-1, Agilent Dako, the fixation conditions, processing, and results evaluation were performed according to international recommendations [35, 36]. TILs were evaluated on H&E-stained slides using standardized methodology [37]. The anti‐cleaved caspase‐3 was assessed using Asp175, Cell Signaling; 1:200; the quantification of cleaved caspase-3, as an experimental parameter, has no international quantification guidelines; by recommendation of expert pathologists, it was performed using QuPath® bio-image analysis software and a H-score = (% of cells with weak intensity × 1) + (% of cells with moderate staining × 2) + (% of cells with strong staining × 3). The maximum possible H-score is 300, corresponding to 100% of cells with strong (3+) intensity.

To evaluate RANK and RANKL, paraffined tissue sections (4 μm) were used. For each patient, representative unstained slides of the tumor were shipped to NeoGenomics Laboratories (California, USA) for IHC staining of RANK (N1H8, Amgen), RL (M366, Amgen), blinded to clinical information. The percentage of stained cells and their intensity (0, negative; 1+, weak; 2+, moderate; and 3+, strong) in the tumor cells were reported by NeoGenomics, and the H-score was calculated using the previous formula. Due to the complexity of RANK and RANKL IHC interpretation and its reading, a double evaluation of H-scores in tumor cells was performed (NeoGenomics and at the laboratory of Dr. Gonzalez-Suarez). In addition, RANK and RANKL H-scores in stroma were evaluated only at the laboratory of Dr. Gonzalez-Suarez [2].

### Serum analysis

Serum concentrations of human free RANKL (sRANKL), tartrate-resistant acid phosphatase 5b (TRACP5b), carboxy-terminal collagen crosslinks (CTX), and OPG were quantified utilizing an enzyme-linked immunosorbent assay (ELISA), in accordance with the manufacturer’s guidelines. Progesterone, estradiol, follicle-stimulating hormone (FSH or Follitropin), calcium, albumin, and blood count values were extracted from laboratory reports processed at the Hospital Universitari de Bellvitge laboratory, where the patient’s routine tests were performed.

### Statistical analysis

The total patient sample size was defined as 60:40 in the treatment arm and 20 in the control arm. Since there were no prior data on the distribution, mean and standard deviation of the primary endpoint available at the time of the protocol design, a conventional statistical design for sample size calculation was not feasible; therefore, the trial was designed according to Larry V. Rubinstein’s suggestions for phase 0 trials [38]. For biological purposes, a minimum of 10 TNBC tumors and a minimum of 24 pre-menopausal patients were included. Assignment to each arm was done by stratified block randomization.

All analyses were performed using R version 4.1.3 (available at www.r-project.org), GraphPad Prism version 5 and IBM SPSS Statistics version 25 (IBM Corp, Armonk, NY, USA). At baseline (biopsy/serum A) *vs.* at surgery (surgery/serum B) values were compared using a paired *t* test or McNemar test for numeric and binary variables, respectively. Independent samples *t* test was used to compare differences between groups, while chi-squared or Fisher exact test, when appropriate, was used for binary variables.

To compare baseline variables and possible predictive factors for response to denosumab, the Mann–Whitney *U* and Fisher’s exact tests were used for continuous and categorical variables, respectively. All correlations were measured using Spearman’s non-parametric rho coefficient. A Logistic regression analysis was performed to define the odds ratio of developing a response variable. All reported *p*-values were two-tailed. Statistical significance was set at 0.05.

## Results

### Patient population

**Fig. 2:**
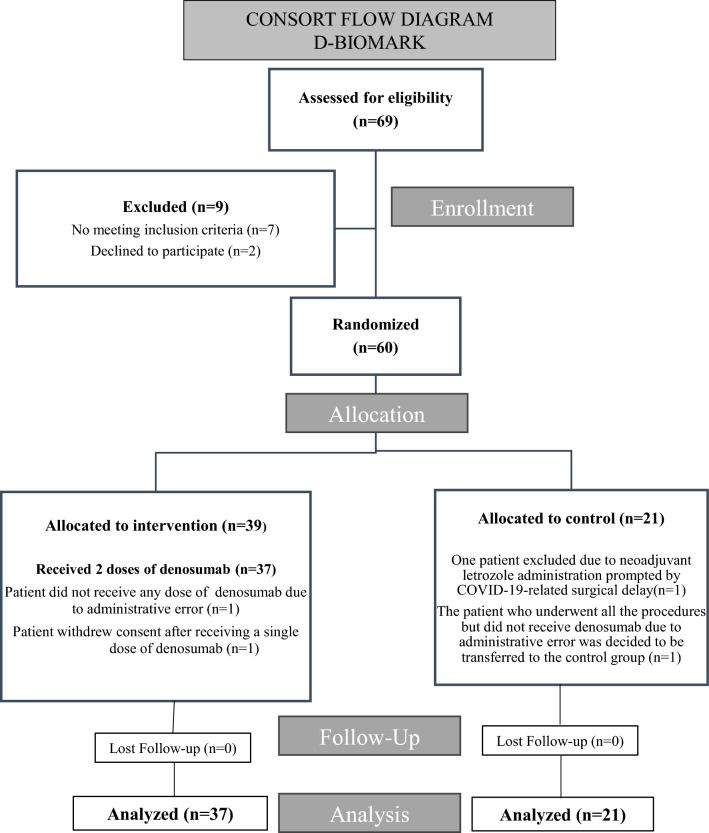
CONSORT Flow diagram. The CONSORT flow chart illustrates the flow of participants throughout the D-BIOMARK study. A total of 60 patients were initially enrolled, with 58 patients evaluated in the final analysis, 37 patients in the experimental arm and 21 patients in the control arm. The analysis was performed according to protocol

A total of 60 patients were enrolled in the study between August 2018 and May 2021. Two patients were excluded. One of them received neoadjuvant letrozole during the SarsCov2 pandemic period due to a delay in the scheduled date of surgery, and other patient only received one dose of denosumab due to withdrawal of consent after the first infusion. One patient who underwent all the procedures but did not receive denosumab due to an administrative error was transferred to the control group. Thus, a total of 37 patients were analyzed in the experimental group and 21 patients in the control group, all 58 patients completed the follow-up period (Fig. 2). The analysis was done by protocol.

The clinicopathological baseline characteristics of the evaluable patients are shown in Table 1. The mean age of the study population was 56.4 years (range, 37–80 years), with a mean age of 57 years in the experimental arm and 55.4 years in the control arm. The mean time between the first administration of denosumab and surgery was 21 days. Of the total participants, 27 were pre-menopausal women, with 17 (45.9%) in the experimental arm and 10 (47.6%) in the control group, meeting the requirement of including at least 24 pre-menopausal women. The distribution of post-menopausal patients was: 20 (54.1%) in the experimental arm and 11 (52.4%) in the control group. Most patients were classified as IA clinical stage, with 26 cases (70.3%) in the experimental arm and 17 cases (81%) in the control arm. The median tumor size was 18 mm (range 8–45 mm).

**T﻿able 1:** Clinicopathological baseline characteristics of the 58 evaluable patients

	Experimental	Control	*p* value
***Patients*** (n = 58)	37	21	
***Mean age*** (range) 56.4 (37–80)	57.0 (37–88)	55.4 (40–80)	0.14
***Menopausal status***			
Pre-menopausal	17 (45.9%)	10 (47.6%)	1.0
Post-menopausal	20 (54.1%)	11 (52.4%)	
***Clinical stage***			
IA	26 (70.3%)	17 (81.0%)	0.74
IB	0 (0%)	0 (0%)	
IIA	8 (21.6%)	4 (19.0%)	
IIB	2 (5.4%)	0 (0%)	
IIIA	1 (2.70%)	0 (0%)	
***Histological subtype***			
Carcinoma NOS (ductal)	21 (56.8%)	15 (71.4%)	0.31
Invasive lobular carcinoma	12 (32.4%)	3 (14.3%)	
Others	4 (10.8%)	3 (14.3%)	
***Histological grade***			
G1	13 (35.1%)	4 (19.0%)	0.43
G2	20 (54.1%)	14 (66.7%)	
G3	4 (10.8%)	3 (14.3%)	
***Ki67***			
< 15	16 (43.2%)	2 (9.5%)	0.01
15–30	12 (32.5%)	14 (66.7%)	
> 30	9 (24.3%)	5 (23.8%)	
***Surrogate molecular subtype***			
Luminal A-like	19 (51.4%)	8 (38.1%)	0.71
Luminal B-like	12 (32.4%)	9 (42.9%)	
TNBC	6 (16.22%)	4 (19.05%)	

NOS, Not otherwise specified (ductal); G, Histological Grade of Nottingham; TNBC, Triple-Negative Breast Cancer

In terms of tumor characteristics, the majority of patients had invasive breast cancer without specific features, classified as no special type (NST) or not otherwise specified (NOS) (ductal), 62.07% overall; with a slightly higher percentage in the control arm (71.4% vs. 56.8% in the experimental group), with a lower number of patients with invasive lobular carcinoma in the control arm (14.3 vs. 32.4% in the experimental arm), although these differences were not significant. Most tumors had a histological grade 2 (58.62%), with 7 tumors (12.06%) classified as grade 3; notably, there was a higher percentage of cases with histological grade 1 tumors (35.1%) in the experimental group compared to the control group (19%). The data are consistent with a higher number of cases with low Ki67 (< 15) in the experimental group (43.2%) compared to the control group (9.5%). Ki67 was the only parameter that showed significance (*p*=0.01) when comparing both groups; however, when analyzing the tumors by surrogate molecular subtype, this difference was not significant, although numerically, there were still more cases of luminal A-like tumors in the experimental arm (51.4% vs. 38.1%). The groups were well balanced as no statistically significant differences between experimental and control arms were observed, except for the percentage of patients with tumors with low Ki67 (< 15%) (Table 1). Of the total of 58 evaluable cases, 48 were luminal tumors (27 luminal A-like and 21 luminal B-like), while 10 were TNBC. Initially, a higher percentage of TNBC cases was expected. However, most TNBC tumors were selected for neoadjuvant treatment and were therefore excluded from our study. Upon reviewing these 10 TNBC cases, only 5 exhibited typical aggressive characteristics (histological grade 2/3, Ki67 > 30%), with 3 of these cases in the control group and 2 in the experimental arm. The remaining cases were low aggressive TNBC, including 3 cases of invasive carcinomas with apocrine differentiation, 2 in the experimental arm and 1 in the control arm: apocrine carcinomas are known to exhibit more indolent behavior compared to typical TNBCs. Additionally, the experimental group had 1 case of lobular carcinoma and 1 case of carcinoma not otherwise specified (NOS) ductal, with Ki67 of 10% and 5%, respectively. Given these factors, caution should be exercised in drawing conclusions about the triple negative subgroup.

### Safety data

Five patients experienced localized hematomas in the breast following study biopsies, which were classified as grade 1 and did not require drainage or special measures; 2 of these cases were in the control group and 3 were in the experimental group. In the experimental arm, the most common adverse events were grade 1 or 2 bone pain occurring 24 hours after infusion in 10 of 37 patients (27.03%), grade 1 asthenia in 4 patients (10.81%), grade 1 pain at the denosumab infusion site in 3 patients (8.10%), grade 1 chills in 2 patients (5.41%), and grade 2 dental infection in 1 patient (2.70%). There were no reported cases of hypocalcemia. No grade 3 toxicities were reported (see Table S1 in the Supplementary Appendix).

Outside the study follow-up period, we observed a long-term event of osteonecrosis of the jaw in a heavy-smoker patient who received denosumab on March 10th and 17th, 2021. This patient was initially reported with a dental infection. Symptoms began one month after the last dose of denosumab (reported on April 19, 2021), with discomfort and pain in the jaw, the case was referred to the Maxillofacial Surgery Department. Computed tomography initially did not reveal signs of osteonecrosis, and the case was initially diagnosed as a tooth infection, which improved with oral antibiotic treatment. However, repeated episodes of dental infection in the same location needed specific follow-up. After 11 months, the patient underwent jaw surgery in February 2022, and the pathological report confirmed osteonecrosis of the right quadrant 44–47. This event was considered possibly related to denosumab, although other triggering risk factors such as chronic infection due to long-term smoking should be considered. The osteonecrosis of the jaw was classified as grade 3. At the time of this report, no grade 4 or 5 toxicity has been reported.

### Serum analysis

#### Denosumab was associated with systemic inhibition of RANKL but not with changes in bone remodeling markers

**Fig. 3:**
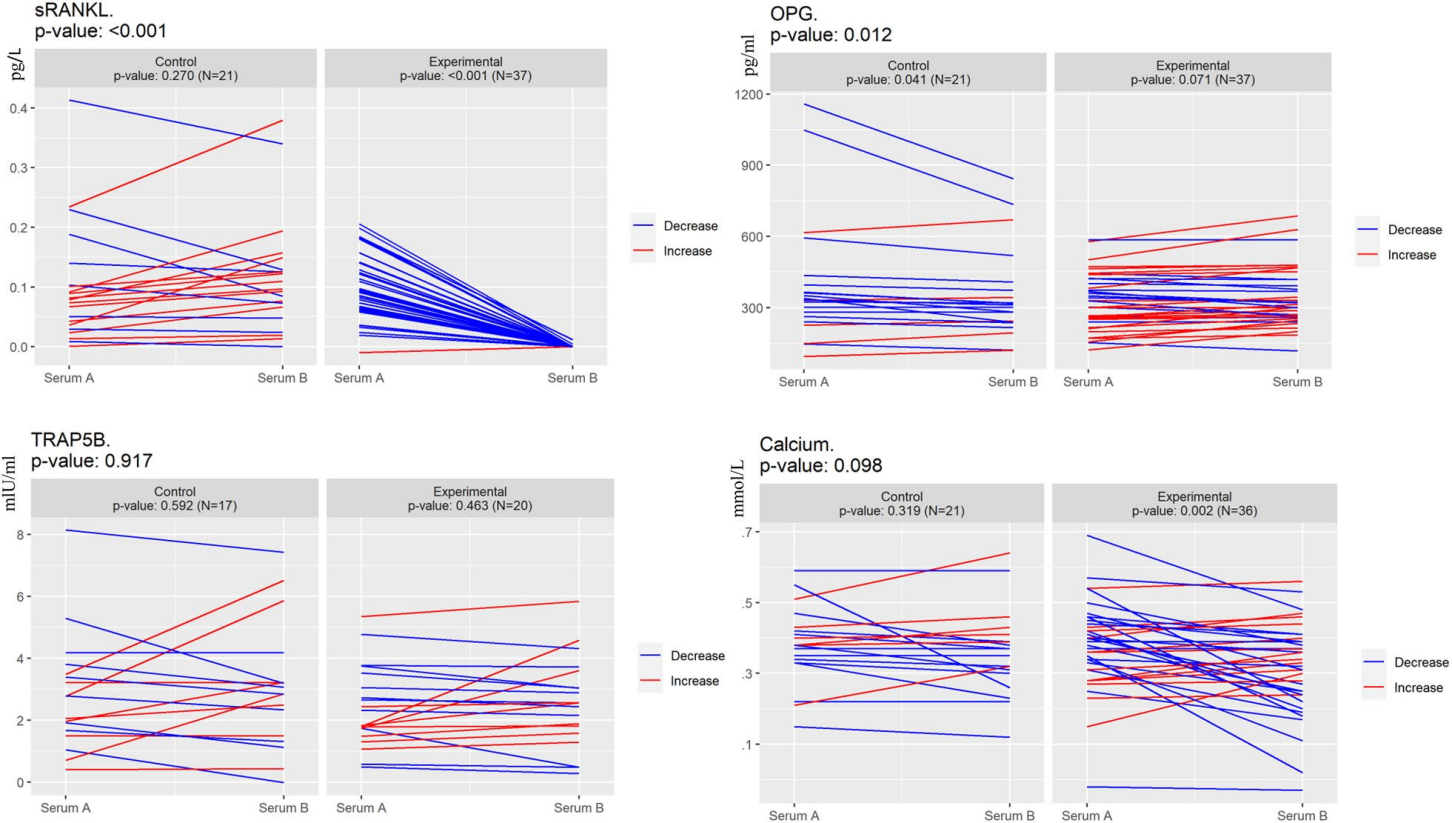
Serum biomarkers: free RANKL (sRANKL), Osteoprotegerin (OPG), Tartrate-resistant acid phosphatase 5b (TRACP-5b) and Calcium. Levels of sRANKL, OPG, TRACP-5b, detected by ELISA, and Calcium in serum from patients collected at the time of biopsy (Serum A) and surgery (Serum B) in the control and experimental arms. *p* value *T* test for comparison between experimental and treatment arm is shown in the upper left corner and *p* value *T* test for paired samples is shown for each treatment group**.** N indicates the number of samples analyzed. Note that Denosumab was associated with reduction in the levels of free RANKL and serum calcium, OPG shows a tendency to increase, while the levels of Trap5b did not change

The blockade of the RANK-RANKL pathway was confirmed by the drop in serum of free RANKL (sRANKL), measured by ELISA, in the experimental group as RANKL became bound to denosumab (mean serum A 0,096 pg./L vs serum B vs. 0,000 pg./L *p*<0.001), while no changes were found in the control group (mean serum A 0,100 pg./L vs. serum B 0,116 pg./L; *p*=0.270) comparing serum A vs serum B. OPG levels tended to increase in the experimental group (*p*=0.071), consistent with the reduction of free RANKL. However, the serum levels of the bone resorption markers** t**artrate-resistant acid phosphatase 5b (TRACP5b) (n=37) and carboxy-terminal collagen crosslinks (CTX) (n=38) did not change in any group (Fig. 3 and Supplementary data Figure S1). It is unclear whether the lack of changes is due to technical limitations (limited detection rate for CTX, Supplementary data Figure S1) or due to the kinetics of bone resorption. Although no significant alterations were observed in bone resorption markers, a small decrease in serum calcium -not clinically relevant- was reported in the experimental arm (Fig. 3), despite the prescribed calcium and vitamin D supplementation. This finding reinforces the efficacy of denosumab in bone remodeling.

A correlation analysis was conducted between the different serum markers studied and menopausal status, to better understand the biology of the pathway. Follicle-stimulating hormone (FSH) levels were higher in post-menopausal patients, consistent with menopausal physiology (*p*<0.0001). Levels of free sRANKL did not differ based on menopausal status, while higher levels of OPG were detected in post-menopausal women (*p*=0.010) at baseline (Supplementary data Figure S2A). Values of the bone markers TRACP5b, and CTX were comparable  at the time of diagnosis (baseline) between pre- and post-menopausal women, although slightly higher levels were found in the post-menopausal group. In pre-menopausal patients, no associations were found between levels of progesterone and sRANKL in serum (*p*=0.401). The correlation between OPG and TRACP5b values was not significant, unless one sample with high OPG and low TRACP5b was excluded. Finally, a negative correlation between OPG and sRANKL was demonstrated (*p*=0.0026), consistent with the known interaction between these factors (Supplementary data Figure S2B).

### Tumor assessment´s results

#### Denosumab was not associated with a reduction in tumor cell proliferation or an increase in apoptosis

**Fig. 4:**
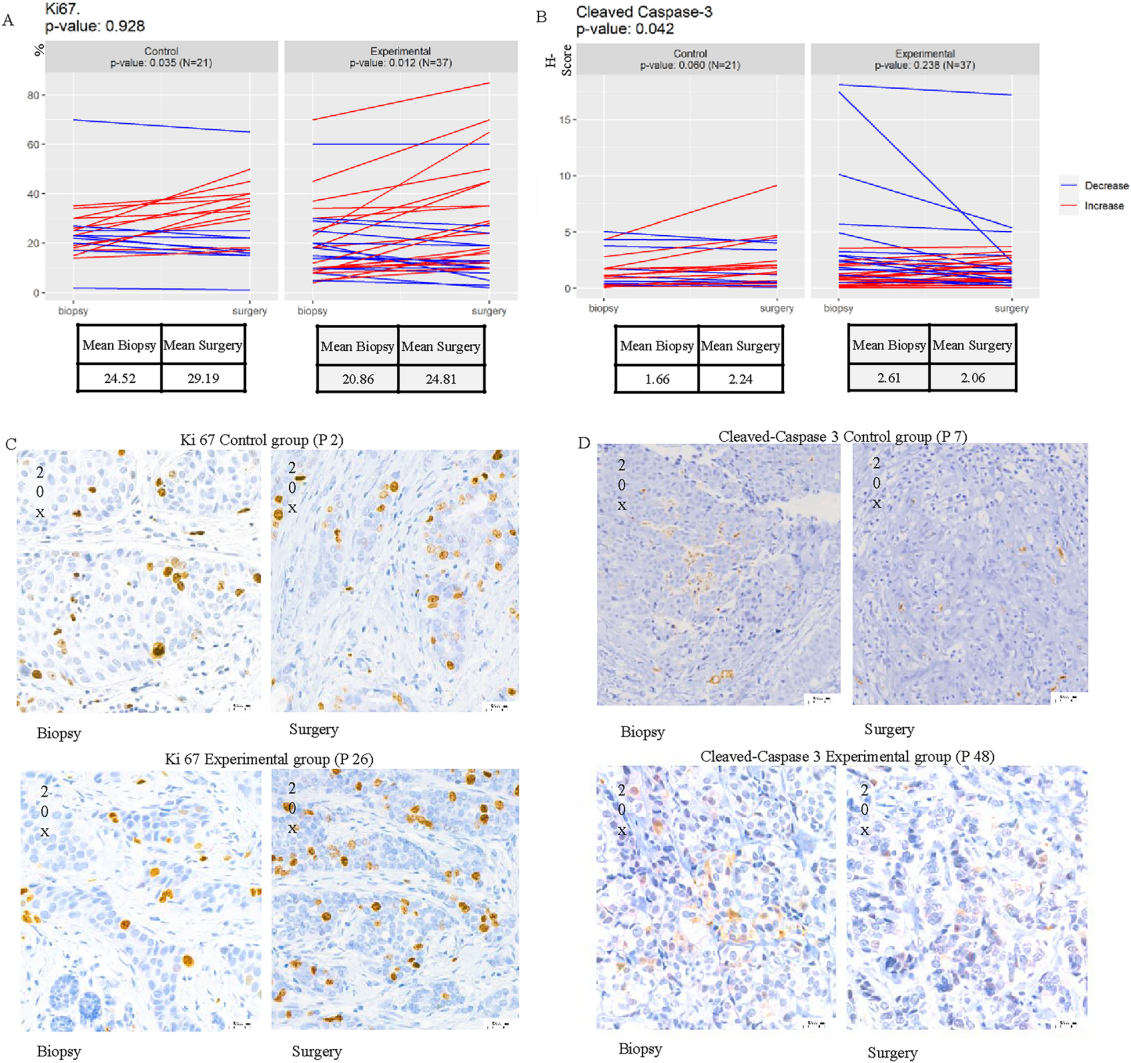
Impact of Denosumab on Tumor Cell Proliferation (Ki67) and apoptosis (cleaved caspase-3). Percentage of Ki-67 + tumor cells (**A**) and H-score of cleaved caspase-3 in tumor cells (**B**) at the diagnostic biopsy (biopsy) and in the surgical specimen (surgery) in the control and experimental arm. *p* value *t* test for comparison between experimental and treatment arm is shown in the upper left corner and *p* value *t* test for paired samples is shown for each treatment group**.** N indicates the number of samples analyzed. **C** and **D** illustrate representative immunohistochemistry staining images from the control and experimental groups for Ki67 and cleaved caspase-3, respectively, P denotes the selected patient. Note that the percentage of Ki67-positive cells in most cases was intermediate (around 20–30%), with a slight, non-clinically relevant increase observed in the surgical specimens. Regarding the apoptosis marker cleaved caspase-3, it was scarcely positive in a few cases, with no changes observed in either group. Comparable mean levels of Ki67 and cleaved caspase-3 were observed between both groups

The primary endpoints of the clinical trial were a decrease in tumor cell Ki67 and an increase in apoptosis between biopsy and surgery. Denosumab did not reduce tumor cell proliferation or survival between paired biopsy and surgery samples (Fig. 4A–D). The percentage of tumor cells expressing Ki67 increased in both groups (control *p*=0.035 and experimental *p*=0.012), which may be attributed to a higher quantification of fields within the surgical specimen (more fields) compared to the core biopsy. The mean Ki67 in the control arm increased from 24.52% at biopsy to 29.19% at surgery. Similarly, in the experimental arm, it increased from 20.86% to 24.81%. This nearly 5-percentage-point increase when comparing surgical samples with baseline biopsies in both groups suggests that these changes were not influenced by denosumab treatment. Indeed, a comparison between the experimental and control groups revealed identical behavior (*p*=0.928) (Figure 4A).

Denosumab did not induce an increase in tumor cell apoptosis, as demonstrated by the assessment of the H-score of cleaved caspase-3 between biopsy and surgery. The comparison between the experimental and control groups revealed notable inter-patient variability. Although a statistically significant difference in apoptosis was observed inter-group (*p*=0.042), the change in cleaved caspase-3 H-score quantification was less than 1 in both groups. Despite the contrasting trends, the alteration in apoptosis is considered clinically insignificant due to very low H-scores in all cases. The evaluating intra-patient (Paired t test) showed no changes (control group *p*=0.060 and experimental *p*=0.238). Additionally, 3 patients in the experimental group exhibited higher levels of cleaved caspase-3 at baseline (Fig. 4B) that may suggest potential deterioration or non-specific staining in some areas.

A subgroup analysis was conducted to elucidate if any specific patient group could benefit from denosumab treatment. Patients were divided according to surrogate molecular subtype (Supplementary data Table S2). Patients with luminal A-like (n=27) and luminal B-like (n=21) tumors showed similar trends to the overall population: there was no reduction in Ki67 or increase in Cleaved Caspase-3 in the experimental group and no changes were observed in OPG or TRACP5b. In the TNBC group (n=10), there was an increase in Ki67 in the experimental arm (*p*=0.025), which was not evident in the control group (*p*=0.517), but no difference was found in the inter-group comparison (*p*=0.197). Moreover, it is important to note the small number of TNBC cases, with 6 in the experimental group and 4 in the control group, as well as an imbalance between aggressive tumors, as previously explained (Supplementary data Table S2).

A subgroup analysis was also conducted based on menopausal status, excluding cases of triple-negative tumors to avoid biasing the information, as 9 out of 10 TNBC tumors were post-menopausal. Both pre-menopausal (n=26) and post-menopausal (n=22) tumors showed similar trends, with no reduction in proliferation or cell survival, and no other notable findings (Supplementary data Table S3).

#### Denosumab increased tumor infiltrating lymphocytes in early breast cancer, particularly in luminal B-Like tumors and regardless of menopausal status

**Fig. 5:**
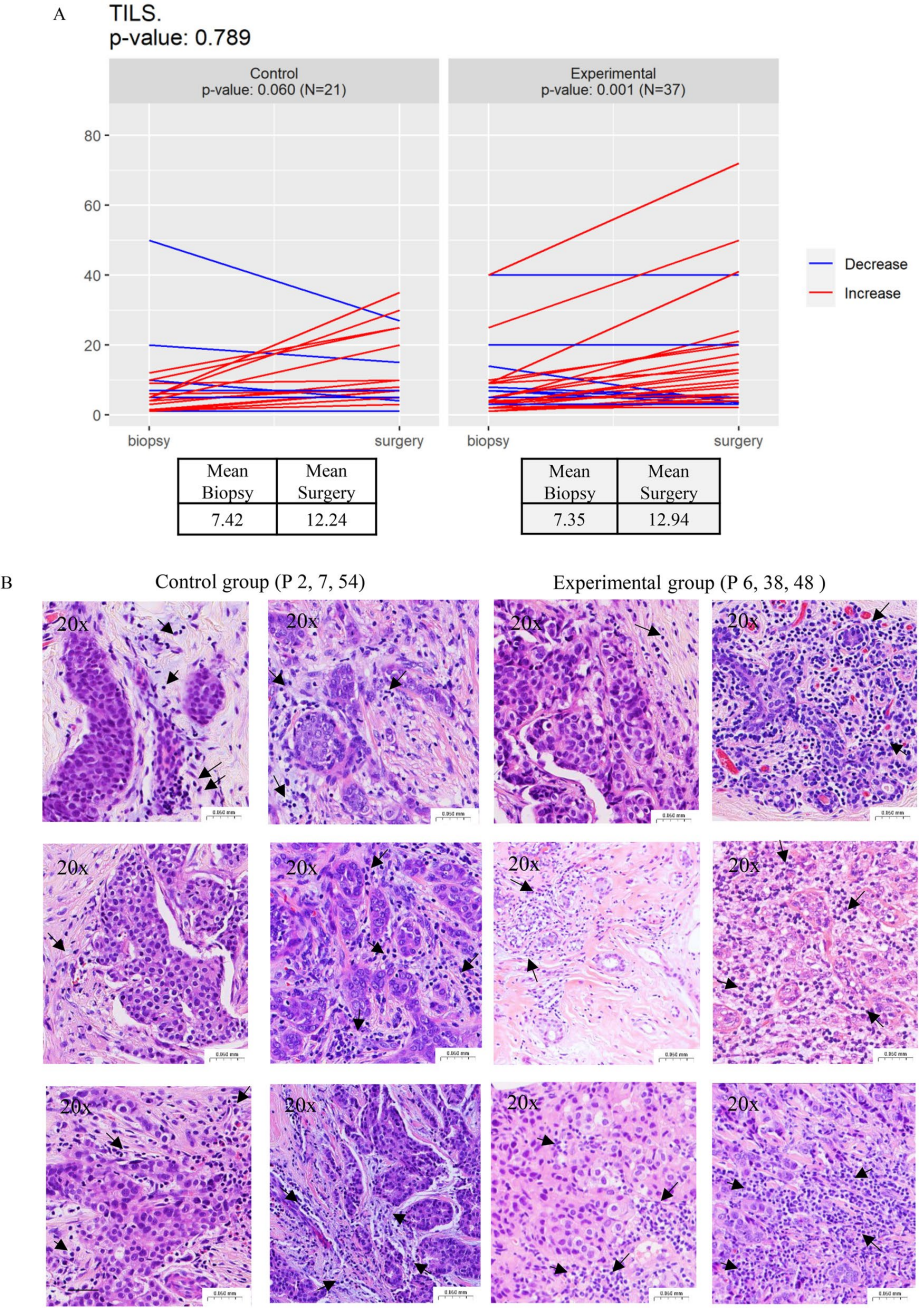
Impact of Denosumab on stromal Tumor Infiltrating Lymphocytes (TILs). **A**: Percentage of stromal TILs at the diagnostic biopsy (biopsy) and in the surgical specimen (surgery) in the control and experimental arm. *p* value *T* test for comparison between experimental and treatment arm is shown in the upper left corner and p value t-test for paired samples is shown for each treatment group**.** N indicates the number of samples analyzed. **B**: Representatives images of H&E staining depicting changes in TILs between biopsy and surgery in patients from the control and experimental arm. Black arrows indicate regions with TILs, P denotes the selected patient. Note that only the experimental group exhibited a statistically significant increase in the percentage of TILs

Next, we interrogated the effect of denosumab on tumor immune infiltration and observed an increase in TILs in the surgery sample compared to the initial biopsy in the experimental arm (*p*=0.001). A similar trend was found in the control arm (*p*=0.06) and indeed, both arms show a similar effect in TILs (*p*=0.789) (Fig. 5A–B).

Performing a similar analysis based on surrogate molecular subtype, we found that while no changes in TILs were noted in Luminal A tumors (experimental (*p*=0.144), control (*p*=0.958)), there was a denosumab-induced elevation in TILs in luminal B-like tumors (*p*=0.012), with no significant changes in the control group (*p*=0.070). TILs did not change in TNBC (experimental (*p*=0.079), control (*p*=0.237)), although an increased number of representative TNBC tumors is required to reach conclusions in this subtype. Attending to menopausal stage in luminal tumors, we found that both pre-menopausal and post-menopausal patients experienced an increase in TILs in the experimental arm (premenopausal: *p*=0.048, postmenopausal: *p*=0.041), but not in the control arm (premenopausal: *p*=0.639, postmenopausal: *p*=0.062). In all comparisons, the experimental and control arms showed similar trends (Table 2) (Supplementary data Figure S3). Furthermore, applying a threshold of a 10% or greater increase in TILs between biopsy and surgery samples, we observed that 9 out of 37 (24.3%) patients in the experimental arm and 5 out of 21 (23.8%) patients in the control arm showed a clinically relevant  increase in TILs.

**Table 2:** Impact of denosumab on stromal tumor-infiltrating lymphocytes (TILs), subgroup analysis by surrogate molecular subtype, and menopausal status

TILs						
***Luminal A-like (N = 27)***						
Control (N = 8)			Experimental (N = 19)			*p*-inter*
Biopsy	Surgery	*p* value	Biopsy	Surgery	*p* value	
10.04	10.25	0.958	4.61	6.23	0.144	0.738
***Luminal B-like (N = 21)***						
Control (N = 9)			Experimental (N = 12)			*p*-inter*
Biopsy	Surgery	*p* value	Biopsy	Surgery	*p* value	
6.50	13.11	0.070	8.55	18.04	**0.012**	0.527
***TNBC (N = 10)***						
Control (N = 4)			Experimental (N = 6)			*p*-inter*
Biopsy	Surgery	*p* value	Biopsy	Surgery	*p* value	
4.25	14.25	0.237	13.67	24.00	0.079	0.969
***ER + Pre-menopausal (N = 26)***						
Control (N = 10)			Experimental (N = 16)			*p*-inter*
Biopsy	Surgery	*p* value	Biopsy	Surgery	*p* value	
10.68	12.60	0.639	7.31	12.72	**0.048**	0.467
***ER + Post-menopausal (N = 22)***						
Control (N = 7)			Experimental (N = 15)			*p*-inter*
Biopsy	Surgery	*p* value	Biopsy	Surgery	*p* value	
4.57	10.57	0.062	4.87	8.75	**0.041**	0.511

Bold indicates statistical significance, defined as *p* < 0.05

*Unpaired T-test for comparison between experimental and control arm

TNBC, Triple-Negative Breast Cancer

#### Tumor and stroma RANK expression was associated with highly proliferative tumors

**Fig. 6:**
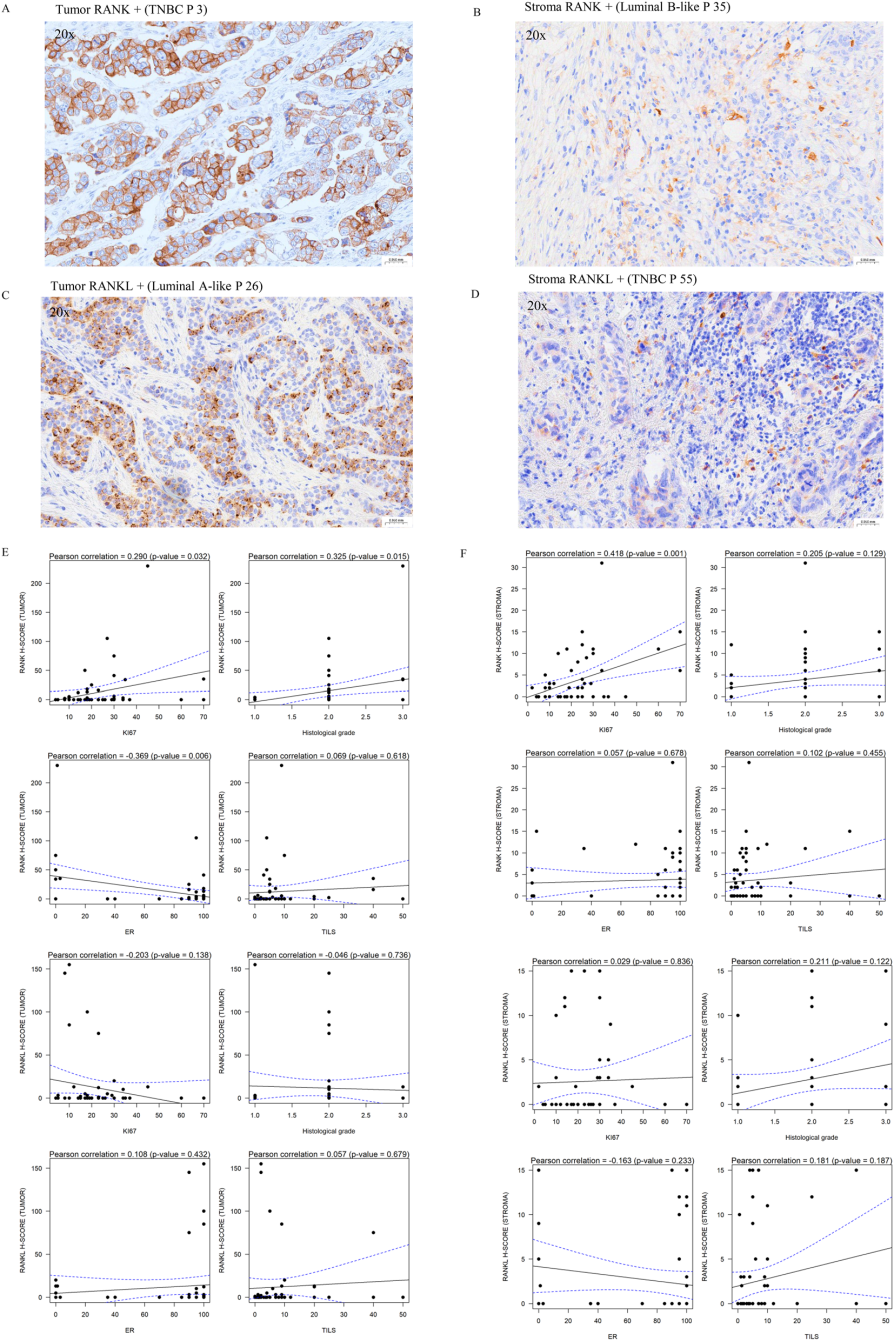
Correlation analysis between RANK/RANKL expression at baseline in tumor or stroma and clinicopathological parameters. **A**: Representative image of RANK expression in tumor cells; **B**: Representative image of RANK expression in stromal cells; **C**: Representative image of RANKL expression in tumor cells; and **D**: Representative image of RANKL expression in stromal cells. The molecular surrogate subtype is indicated in parentheses, P denotes the selected patient. **E**: Correlation analysis between RANK/RANKL expression in tumor with Ki67, histological grade, ER and TILs using Pearson’s correlation coefficient. **F**: Correlation analysis between RANK/RANKL expression in stroma with Ki67, histological grade, ER and TILs using Pearson’s correlation coefficient. Note that RANK expression in tumor cells positively correlates with Ki67, histological grade, and negatively correlates with estrogen receptor expression. Additionally, RANK expression in stroma is associated with high Ki67 levels. No significant correlation was found with RANKL expression

A total of 55 (95%) cases were assessable for tumor RANK and RANKL expression at baseline (Table 3, Fig. 6). Representative images of RANK/RANKL expression in tumor and stromal cells are shown in Fig. 6A–D. The quantification conducted externally by NeoGenomics and in-house by the laboratory of Dr. Gonzalez-Suarez showed perfect correlation in the analysis of tumor RANK and RANKL protein expression (Supplementary data Figure S4). Given the correlation and the fact that the quantification of H-score for RANK and RANKL in the stroma was only conducted in the laboratory of Dr. Gonzalez-Suarez, all subsequent analyses were carried out using in-house quantification.

**Table 3:** Expression of RANK and RANKL in tumor cells and stroma at baseline biopsy, and its relationship with treatment arm, menopausal status, surrogate molecular subtype, and histological grade

	Tumor RANK			Tumor RANKL			Stroma RANK			Stroma RANKL		
	Tumor RANK-	Tumor RANK +	*p* value	Tumor RANKL-	Tumor RANKL +	*p* value	Stroma RANK-	Stroma RANK +	*p* value	Stroma RANKL-	Stroma RANKL +	*p* value
	N = 36	N = 19		N = 38	N = 17		N = 29	N = 27		N = 37	N = 18	
***Treatment***			0.526			0.819			0.632			0.569
Experimental	22 (61.1%)	14 (38.9%)		24 (66.7%)	12 (33.3%)		20 (55.6%)	16 (44.4%)		25 (71.4%)	10 (28.6%)	
Control	14 (73.7%)	5 (26.3%)		14 (73.7%)	5 (26.3%)		9 (45.0%)	11 (55.0%)		12 (60.0%)	8 (40.0%)	
***Menopausal status***			1,000			1,000			0.292			1,000
Pre-menopausal	16 (64.0%)	9 (36.0%)		17 (68.0%)	8 (32.0%)		11 (42.3%)	15 (57.7%)		17 (68.0%)	8 (32.0%)	
Post-menopausal	20 (66.7%)	10 (33.3%)		21 (70.0%)	9 (30.0%)		18 (60.0%)	12 (40.0%)		20 (66.7%)	10 (33.3%)	
***Surrogate molecular subtype***			0.460			0.861			**0.012**			0.284
Luminal A-like	18 (72.0%)	7 (28.0%)		18 (72.0%)	7 (28.0%)		17 (65.4%)	9 (34.6%)		20 (76.9%)	6 (23.1%)	
Luminal B-like	13 (65.0%)	7 (35.0%)		14 (70.0%)	6 (30.0%)		5 (25.0%)	15 (75.0%)		12 (63.2%)	7 (36.8%)	
TNBC	5 (50.0%)	5 (50.0%)		6 (60.0%)	4 (40.0%)		7 (70.0%)	3 (30.0%)		5 (50.0%)	5 (50.0%)	
***Histological grade***			0.137			0.308			0.215			0.422
G1	13 (86.7%)	2 (13.3%)		12 (80.0%)	3 (20.0%)		11 (68.8%)	5 (31.2%)		13 (81.2%)	3 (18.8%)	
G2	19 (57.6%)	14 (42.4%)		20 (60.6%)	13 (39.4%)		14 (42.4%)	19 (57.6%)		20 (62.5%)	12 (37.5%)	
G3	4 (57.1%)	3 (42.9%)		6 (85.7%)	1 (14.3%)		4 (57.1%)	3 (42.9%)		4 (57.1%)	3 (42.9%)	

Bold indicates statistical significance, defined as *p* < 0.05

G, Histological Grade of Nottingham; TNBC, Triple-Negative Breast Cancer

As shown in Table 3, a total of 19 tumors (34.5%) exhibited positive baseline expression of RANK, defined as an H-score > 0 (tumor RANK+). Of these positive cases, 14 tumors were randomized to the experimental arm (38.9%), and 5 were assigned to the control group (*p*=0.526, well balanced). The frequency of RANK+ tumors was comparable between tumors from pre-menopausal (36%) and post-menopausal patients (33%). Additionally, we compared tumor RANK expression across different molecular subtypes, 28% of luminal A-like, 35% of luminal –B-like and 50% of triple negative tumors exhibited RANK+ tumor cells, a higher frequency than that previously reported, particularly in luminal tumors [18, 19]. A total of 42.4% of grade 2 and 42.9% of grade 3 tumors were positive for tumor RANK, compared to only 13.3% of grade 1 tumors.

Regarding RANKL expression, 17 out of 55 tumors (30.1%) exhibited RANKL expression in tumor cells, with 12 assigned to the experimental group and 5 to the control group (*p*=0.819, well balanced). The frequency of tumor RANKL expression was similar between pre- and post-menopausal conditions (32% and 30%, respectively). No differences were found concerning molecular subtype or histological grade (Table 3). In only 8 samples (14.5%), both RANK+ and RANKL+ tumor cells were identified, but in these overlapping cases H-scores were low.

Based on the assessment of RANK and RANKL expression in stromal cells, 27 out of 56 evaluable samples (48,2%) and 18 out of 55 cases (32.7%) respectively exhibited an H-Score>0. Both the control and experimental groups were well balanced at baseline. There were no differences based on menopausal status; although in the pre-menopausal group stromal RANK expression was found in 57.7% compared to 40% in post-menopausal. Upon analysis by molecular subtype, it was noteworthy that luminal B-like tumors exhibited elevated RANK expression in the stroma, with rates of 75% compared to 34.5% in luminal A-like tumors and 30% in TNBC. This difference was statistically significant (*p*=0.012), representing the sole parameter where such distinction was observed (Table 3).

Observing this pattern and recognizing that RANK and RANKL expressions behave more like continuous than categorical variables, we decided to perform an analysis between Ki67, histological grade, ER expression and TILs at the level of basal biopsies (to avoid deviations related to denosumab) and correlate them with the IHC expression of RANK and RANKL in these biopsies (Fig. 6E–F). A positive correlation was identified between RANK expression in tumor cells and cell proliferation (Ki67) p=0.03, and histological grade *p*=0.015, while a negative correlation with ER expression *p*=0.006 and no association with % of TILs was observed (Fig. 6E). The findings indicated that cases characterized by high histological grade, high Ki67 levels, and low estrogen receptor expression showed higher expression of RANK protein in tumor cells (Fig. 6E). Despite tumors with the highest RANKL scores (H > 25) showed low levels of ki67, RANKL expression in tumor cells did not associate with Ki67, nor with the other parameters (Fig. 6E). Strikingly, RANK expression in the stroma was associated with high Ki67 expression *p*=0.001, while stromal RANKL did not associate with any parameter (Fig. 6F). The percentage of TILs at baseline did not associate with RANK or RANKL expression. The notable correlation observed in between RANK expression in both tumor and stroma underlines its association with aggressive tumors.

When restricting the analyses to luminal tumors neither RANK nor RANKL expression in tumor cells associated with any of the parameters analyzed (Supplementary data Figure S5A-B). Notably, increased stromal RANK expression remained associated with high Ki67 levels (*p*=0.001) and a positive correlation between RANKL in the stroma and TILs was observed (possibly as a marker of this population) (Supplementary data Figure S5C-D). Therefore, we can infer that higher tumor proliferation correlates with higher RANK expression in the stroma in luminal tumors and the global analyses including TNBC. These results provide valuable information on the relationship between RANK, RANKL and other BC markers.

As expected, no changes in the expression of RANK or RANKL in tumor cells or in the stroma were found between biopsy and surgery, neither in the control nor in the experimental arm (Supplementary data Figure S6).

#### Baseline RANK or RANKL expression did not predict denosumab-driven changes in TILs

Univariate and multivariate analyses were performed to identify possible factors associated with the 10% increase in TILs. Only having a high Ki67 (>30) could be related to an elevation in TILS, despite no reaching significance in the multivariate analyses (OR 7.12 (1.18-43.1) (*p*=0.079) (Table 4). The only significant factor identified in the multivariate analysis that correlated with an increase in TILs was RANK expression in tumor cells at baseline (*p* < 0.001). No other factors were found to be significantly associated with the 10% increase in TILs (Table 4).

**Table 4:** Univariate and multivariate analysis on increase in TILs ≥ 10% as response variable

	N	No event(%)	Event(%)	Chi-Square p-value	Univariate OR (95%CI)	*p* value	OR mult	*p* value
***Treatment arm***								
Control	21	16 (76.2%)	5 (23.8%)	1	Ref	Ref	Ref	0.962
Experimental	37	28 (75.7%)	9 (24.3%)	–	1.03 [0.29;3.60]	0.978	0.68	
***Ki67***								
≤ 15%	21	19 (90.5%)	2 (9.52%)	0.075	Ref	Ref	Ref	0.079
16–29%	23	17 (73.9%)	6 (26.1%)	–	3.35 [0.60;18.9]	0.183	1.55	
≥ 30%	14	8 (57.1%)	6 (42.9%)	–	7.12 [1.18;43.1]	0.033	2.81	
***Menopausal status***								
Pre-menopausal	27	19 (70.4%)	8 (29.6%)	0.545	Ref	Ref	Ref	0.22
Post-menopausal	31	25 (80.6%)	6 (19.4%)	–	0.57 [0.17;1.92]	0.384	0.21	
***Surrogate molecular subtype***								
Luminal A-like	27	24 (88.9%)	3 (11.1%)	0.089	Ref	Ref	Ref	0.336
Luminal B-like	21	14 (66.7%)	7 (33.3%)	–	4.00 [0.89;18.0]	0.076	1.4	
TNBC	10	6 (60.0%)	4 (40.0%)	–	5.33 [0.93;30.5]	0.078	0.13	
***Histological Subtype***								
Carcinoma NOS (ductal)	14	11 (78.6%)	3 (21.4%)	0.742	Ref	Ref	Ref	0.512
Invasive lobular carcinoma	37	27 (73.0%)	10 (27.0%)	–	1.36 [0.31;5.90]	0.717	1.02	
Others	7	6 (85.7%)	1 (14.3%)	–	0.61 [0.05;7.24]	0.76	0.01	
***Histological grade***								
G1	17	15 (88.2%)	2 (11.8%)	0.061	Ref	Ref	Ref	0.964
G2	34	26 (76.5%)	8 (23.5%)	–	2.31 [0.43;12.3]	0.353	0.34	
G3	7	3 (42.9%)	4 (57.1%)	–	10.0 [1.22;81.8]	0.041	1.5	
RANK/ RANKL H-Scores (Median [1Q; 3Q])*								
RANK TUMOR	55	0.00 [0.00;2.00]	7.50 [0.00;34.8]	**0.012**	1.05 [1.01;1.09]	**0.026**	1.15	**< 0.001**
RANKL TUMOR	55	0.00 [0.00;2.00]	0.00 [0.00;4.50]	0.612	1.00 [0.98;1.02]	0.821	0.99	0.455
RANK STROMA	56	0.00 [0.00;3.00]	4.50 [0.00;10.5]	0.147	1.06 [0.96;1.17]	0.225	0.99	0.967
RANKL STROMA	55	0.00 [0.00;2.00]	1.00 [0.00;8.00]	0.115	1.08 [0.96;1.22]	0.182	1.11	0.261

Bold indicates statistical significance, defined as *p* < 0.05

^*^For RANK/RANKL H-scores, Mann–Whitney test has been applied

NOS, Not otherwise specified (ductal); G, Histological Grade of Nottingham

Finally, when we analyzed the expression of RANK and RANKL as possible biomarkers of response to denosumab, we observed that the trends were similar to the overall population (Tables 5 and 6). There was no reduction in Ki67 or increase in cleaved caspase-3 after denosumab treatment when the analyses were performed only in tumors expressing RANK or RANKL protein in the tumor or stroma at baseline. Importantly, denosumab increased TILs regardless of tumor and stroma RANKL or RANK expression. This is, in tumor RANK positive samples, the experimental group showed an increase in TILs (*p*=0.013), similar to that observed in tumors that did not express RANK (tumor RANK-) (*p*=0.008). The same was observed when tumor RANKL expression was considered, the tumor RANKL positive group showed an increase in TILs (*p*=0.048), similar to the tumor RANKL negative group (*p*=0.002) (Table 5). The benefit of increased TILs after denosumab treatment was observed in tumors, irrespectively of the stromal expression of RANK or RANKL (Table 6). In conclusion, RANK and RANKL protein expression at baseline cannot be used as a biomarker capable of predicting the elevation of TILs caused by denosumab.

**Table 5:** Analysis of tumor cell protein expression of RANK and RANKL across different response variables

	*Tumor RANK- (N = 36)*							*Tumor RANK + (N = 19)*						
	Control (N = 14)			Experimental (N = 22)				Control (N = 5)			Experimental (N = 14)			
	Biopsy	Surgery	*p* value	Biopsy	Surgery	*p* value	p-inter*	Biopsy	Surgery	*p* value	Biopsy	Surgery	*p* value	p-inter*
Ki67 (%)	25.71	32.00	0.041	17.95	20.68	0.147	0.292	26.20	29.40	0.384	25.43	33.50	0.062	0.359
Cleaved caspase-3 (H-Score)	1.17	2.03	0.041	1.70	1.59	0.701	0.050	3.25	3.42	0.751	4.20	2.88	0.262	0.243
TILs (%)	8.99	12.79	0.211	3.50	8.33	**0.008**	0.761	3.80	14.60	0.120	12.93	20.88	**0.013**	0.658
sRANKL (pg/L)	0.07	0.11	0.049	0.09	0.00	**0.000**	**0.000**	0.17	0.13	0.184	0.10	0.00	**0.000**	0.059
OPG (pg/ml)	469.14	406.96	0.063	309.41	319.89	0.351	**0.039**	256.39	236.59	0.184	347.73	368.44	0.091	**0.034**
TRACP5b (mlU/ml)	3.00	3.31	0.532	2.17	2.45	0.276	0.959	2.99	2.87	0.811	2.77	2.72	0.892	0.902

	*Tumor RANKL- (N = 38)*							*Tumor RANKL + (N = 17)*						
	Control (N = 14)			Experimental (N = 24)				Control (N = 5)			Experimental (N = 12)			
	Biopsy	Surgery	*p* value	Biopsy	Surgery	*p* value	p-inter*	Biopsy	Surgery	*p* value	Biopsy	Surgery	*p* value	p-inter*
Ki67 (%)	26.21	34.43	0.006	22.00	24.62	0.146	0.081	24.80	22.60	0.307	18.58	27.75	0.063	0.033
Cleaved caspase-3 (H-Score)	1.57	2.42	0.053	2.87	2.18	0.323	0.060	2.12	2.35	0.599	2.29	1.92	0.401	0.325
TILs (%)	7.91	14.21	0.082	5.92	11.94	**0.002**	0.941	6.80	10.60	0.330	9.67	15.75	**0.048**	0.615
sRANKL (pg/L)	0.08	0.09	0.396	0.10	0.00	**0.000**	**0.000**	0.16	0.18	0.605	0.08	0.00	0.001	0.046
OPG (pg/ml)	414.20	370.73	0.087	325.35	341.53	0.108	**0.031**	410.23	338.03	0.307	322.22	333.24	0.466	0.253
TRACP5b (mlU/ml)	2.95	3.31	0.395	2.43	2.27	0.079	0.234	3.15	2.86	0.768	2.39	3.00	0.211	0.413

Bold indicates statistical significance, defined as *p* < 0.05

^*^T test for non-paired samples used for comparison between the experimental and control arms

**Table 6:** Analyses of stromal protein expression analysis of RANK and RANKL across different response variables.

	*Stroma RANK- (N = 29)*							*Stroma RANK + (N = 27)*						
	Control N = 9			Experimental N = 20				Control N = 11			Experimental N = 16			
	Biopsy	Surgery	*p* value	Biopsy	Surgery	p-value	p-inter*	Biopsy	Surgery	*p* value	Biopsy	Surgery	*p* value	p-inter*
Ki67 (%)	21.00	26.11	0.160	17.40	24.65	0.020	0.629	27.64	32.82	0.100	25.19	26.94	0.458	0.360
Cleaved caspase-3 (H-Score)	1.89	2.36	0.203	1.52	1.61	0.740	0.389	1.46	2.23	0.137	4.12	2.70	0.171	0.058
TILs (%)	11.11	15.00	0.438	6.08	10.16	**0.004**	0.969	4.98	11.00	**0.046**	8.54	17.02	**0.009**	0.531
sRANKL (pg/L)	0.14	0.15	0.622	0.08	0.00	**0.000**	**0.008**	0.06	0.08	0.244	0.12	0.00	**0.000**	**0.000**
OPG (pg/ml)	296.58	283.44	0.226	357.44	376.50	0.114	0.045	503.95	422.51	0.051	282.90	291.60	0.440	**0.037**
TRACP5b (mlU/ml)	2.22	2.42	0.662	1.43	1.84	0.398	0.739	3.60	3.80	0.744	3.07	3.04	0.815	0.713

	*Stroma RANKL- (N = 37)*							*Stroma RANKL + (N = 18)*						
	Control N = 12			Experimental N = 25				Control N = 8			Experimental N = 10			
	Biopsy	Surgery	*p* value	Biopsy	Surgery	*p* value	p-inter*	Biopsy	Surgery	*p* value	Biopsy	Surgery	*p* value	p-inter*
Ki67 (%)	26.58	30.83	0.204	18.84	21.84	0.140	0.739	21.75	28.25	0.036	25.50	33.30	0.123	0.807
Cleaved caspase-3 (H-Score)	1.42	1.89	0.115	2.60	1.95	0.326	0.120	2.01	2.88	0.213	3.10	2.63	0.399	0.127
TILs (%)	8.88	11.42	0.387	5.82	11.22	**0.002**	0.385	6.04	14.88	0.096	10.55	19.00	**0.046**	0.948
sRANKL (pg/L)	0.09	0.11	0.360	0.10	0.00	**0.000**	**0.000**	0.10	0.12	0.527	0.08	0.00	**0.001**	**0.003**
OPG (pg/ml)	433.69	365.64	0.070	309.12	317.85	0.365	0.049	376.05	351.36	0.213	373.50	394.68	0.182	0.068
TRACP5b (mlU/ml)	2.42	2.93	0.339	2.35	2.73	0.223	0.828	4.27	3.78	0.022	2.53	2.32	0.307	0.252

Bold indicates statistical significance, defined as *p* < 0.05

^*^T test for non-paired samples used for comparison between the experimental and control arms

## Discussion

The D-BIOMARK trial was designed to investigate the biological effects of denosumab in patients with HER2-negative early breast cancer as a proof of concept for its potential antiproliferative, proapoptotic, and immunomodulatory effects on these tumors and their microenvironment, beyond its bone-related effects. Our findings are consistent with those of the D-BEYOND trial, which had a similar design but involved a smaller patient cohort, a single treatment arm, and exclusively pre-menopausal women [29]. The D-BIOMARK study overcomes these limitations by incorporating a control arm, encompassing both pre and postmenopausal patients, and including a larger number of TNBC patients. Preoperative denosumab however, did not reduce tumor cell proliferation or cell survival in early breast cancer. Subgroup analyses attending to menopausal status, surrogate molecular subtype and ER expression led to similar conclusions. Notably, we observed a possible effect on the immune response, with a statistically significant increase in TILs in the experimental arm, in pre-menopausal but also in post-menopausal patients, especially in the luminal B-like tumor subgroup.

It is important to note that the RANK signaling has been identified as a crucial pathway in tumor initiation, inducing proliferation in normal epithelium and hyperplasia, but not in advanced lesions (ductal carcinoma in situ and adenocarcinomas) [2]. These data are consistent with negative clinical results from D-BIOMARK and D-BEYOND regarding Ki67 and cleaved caspase-3. Once the tumor is established, the anti-proliferative and pro-apoptotic effects may be lost; however, there must be a mechanism, yet unclear, for those tumors with pharmacological or genetic inhibition of the pathway in preclinical studies to have fewer metastases [11, 12]. This therapeutic effect has also been reflected in clinical practice, with the large adjuvant trial ABCSG-18 demonstrating the benefit of adding denosumab in progression-free survival, bone metastasis-free survival, and overall survival, a trial designed exclusively in the luminal population [23]. This benefit may be related to an anti-tumoral immune activation generated by denosumab, a hypothesis to be considered.

The D-BIOMARK trial observed an increase in TILs following denosumab treatment. Although no significant increase in TILs was observed in the control group, a similar trend was noted. The inflammatory effect induced by the biopsy, differences between biopsy and surgical specimens, or cohort size may have contributed to these variations. However, it is important to highlight that, even in the subgroup analyses, the increase in TILs reached significance only in denosumab-treated patients despite the lower number of samples. Although the increase in TILs was modest to have clinical relevance, it should be noted that patients received only two doses of denosumab one week apart. A limitation of our study was the imbalance due to the 2:1 randomization, resulting in fewer patients in the control group. This issue should be addressed in future trials with larger randomized studies and balanced cohorts. Additionally, further studies should characterize potential changes in the composition of the tumor immune microenvironment to properly assess the immunomodulatory effects of denosumab.

An important implication of these results is the potential use of denosumab as a possible enhancer of immune infiltration in neoadjuvant therapy for luminal B-like tumors, where immunotherapy with pembrolizumab (KEYNOTE-756 clinical trial) or nivolumab (CheckMate 7FL clinical trial) has shown to improve pCR but with rates close to 25%, much lower than in TNBC [39, 40]. Could denosumab improve these results? During neoadjuvant chemotherapy, initially elevated levels of TILs correlate positively with a higher rate of achieving a pCR in all breast cancer subtypes [41]. A booster given by denosumab allowing new combinations of treatments in a tumor microenvironment that would otherwise be cold is of clinical interest. The GeparX study already combined denosumab with neoadjuvant chemotherapy without reporting additional benefits. Although an immediate impact on pCR may not be evident, the possibility of a long-term effect cannot be ruled out [25–28], and perhaps the best partner is the immunotherapy. There are already preclinical data showing synergy between immune checkpoint inhibitors (ICIs) and inhibitors of RANK signaling in solid tumors [29]. The safety of the combination in clinical practice was reported in the CHARLI trial, a phase I/II study of the effect of denosumab with nivolumab (an anti-PD-1), with or without ipilimumab (anti-CTLA4), in patients with metastatic melanoma, which showed that the combination is safe and with at least interesting response rates [42]. Pembrolizumab and denosumab have also been tested in clear cell renal cell carcinoma, a phase II trial (KeyPAD trial) with response rates close to 31% [43], and there are other ongoing trials with ICI in different tumors such as in lung cancer (Popcorn Trial) [44]. Testing the combination in BC will be of interest, although we recognize that more data is required.

Additionally, we have reported the expression of RANK and RANKL in tumor cells and stroma. A total of 34.54% of cases expressed RANK and 30% RANKL in the core biopsy, defined as an H-score > 0, slightly higher than reported in the literature, particularly in luminal tumors [18, 19]. In line with prior studies, RANK expression determined by IHC in tumor cells was associated with ER-negative tumors and high proliferative capacity [17–19]. Our data confirm that RANK protein expression in tumor cells could serve as a biomarker for tumor aggressiveness: a correlation was observed between high Ki67, low ER expression, and high histological grade. Recent data from the GeparX clinical trial showed that RANK expression in tumor cells was an independent predictive biomarker of response to neoadjuvant chemotherapy in luminal breast cancer, highlighting the opposite in TNBC and HER2-positive tumors [28]. Apparently, and according to our data, luminal tumors might also have the RANK pathway involved in their pathophysiology, not only TNBC, where the biological effect of RANK has been reported [18, 19, 45].

Strikingly our data reveals a novel association between RANK protein expression in the tumor microenvironment (stroma) with high levels of tumor proliferation (Ki67), which remains when studying exclusively luminal tumors (*p*=0.001). RANK expression in immune cells is predominantly found in myeloid cells such as macrophages and dendritic cells, while RANKL is predominantly found in TILs [20]. Indeed, RANKL expression in the stroma of luminal tumors was associated with high percentage of TILs. Whether the highly proliferative tumors with RANK+ in the stroma have a greater immunosuppressive immune infiltrate is a hypothesis to be tested in the future.

We were unable to establish a relationship between RANK or RANKL expression, neither in the stroma nor in the tumor cells, as a predictive marker for response to denosumab. However, RANK IHC in tumor cells was the only parameter in the multivariate analysis related to an increase of >10% in TILs between biopsy and surgery. This suggests that RANK expression in tumor cells may serve as a marker for aggressive tumors capable of recruiting higher levels of TILs. The difficulty in detecting RANK or RANKL as a response biomarker may be attributed to the variability and lack of standardization in the immunohistochemical technique for this staining, as well as the lack of a standardized cutoff point. In addition, the response, as evidenced by an increase in TILs after only two doses, was not strong enough to classify the change as a response, making it impossible to identify a biomarker.

Regarding serum markers, the reduction in free sRANKL following denosumab administration confirms effective RANK pathway inhibition in our study. However, the absence of significant changes in TRACP5b and CTX contrasts with previous studies [29, 46, 47]. Technical challenges likely contributed to this discrepancy: CTX measurements have been reported to be poorly reproducible and particularly sensitive to food intake [48, 49]. In our analysis many CTX samples fell below the detection limit and we cannot ensure that they were collected under fasting conditions. Two other parameters may likely limit the detection of changes in bone remodeling markers: the shorter sampling interval in our study (14–21 days post-treatment) compared to prior studies months-long intervals [46, 47], and the fact that 47% of our cohort were premenopausal with lower baseline levels of bone resorption markers when compared to osteoporotic women or patients with bone metastasis. Despite these limitations, the reduction in free sRANKL and serum calcium levels in the experimental group supports Denosumab’s bone-modulatory effects, even though bone turnover markers were not a primary focus of this trial. Additionally, there was an increase in OPG in the experimental group, related to the decrease in sRANKL, suggesting an incremental feedback loop or that denosumab binding to RANKL "displaces" OPG, increasing the detection of free OPG in circulation. Our analysis revealed a significant negative correlation between both markers. Surprisingly, post-menopausal women in our study displayed higher OPG levels, diverging from what is reported in the literature [50]. The complexity of interpreting biomarker dynamics is underscored by factors such as bone mineral density, body mass index, and cardiovascular disease, which were not collected at enrollment [51, 52]. Additionally, sRANKL levels showed no association with age or menopausal status. These nuanced findings emphasize the need for comprehensive data collection to unravel the multifaceted influences on this pathway.

As limitations of our study, we can highlight the small sample size limiting subgroup analyses, the statistical imbalance due to the 2:1 randomization and the tumor heterogeneity, especially affecting the cohort of TNBC, with a clear selection bias since most of these tumors receive neoadjuvant chemotherapy. This subgroup only includes 5 out of 10 typical aggressive TNBC, 2 cases with low proliferation index tumors and 3 cases of apocrine neoplasms. As strengths of our proposal, unlike the D-BEYOND study [29], we included a control group that revealed the potential inflammatory effect of the biopsy. Moreover, the inclusion of a higher number of samples allowed us not only to generate hypotheses regarding the role of denosumab as an immune modulator in premenopausal women, but also to extend these findings to postmenopausal women. This study provides clinical validation of preclinical observations, contributing to a better understanding of the biological effects of this drug and paving the way for the design of future trials.

Consequently, our study serves as a crucial tool for understanding the behavior of this pathway within breast tumor cells, tumor microenvironment, and serum markers and opens new hypotheses for the implications of denosumab as a therapeutic target in BC, beyond its bone-related effects. This knowledge is essential for developing new drugs and for the more efficient utilization of denosumab.

## Conclusions

Two doses of denosumab before surgery did not reduce tumor proliferation or increase tumor apoptosis. A short course of denosumab could increase TILs in early breast cancer, particularly in luminal B-like tumors, and in pre-and postmenopausal women with breast cancer.

These findings suggest that denosumab may enhance the body’s immune response against breast cancer, paving the way for further exploration and treatment refinement. Moreover, this trial underscores the complex nature of the RANK pathway, highlighting the necessity for further investigation into its multifaceted aspects.

## Supplementary Information

Additional file 1.

## Data Availability

All data and histology images supporting the findings of this study are fully available and digitally stored. Due to sensitivity reasons, they are not openly accessible but can be obtained from the corresponding author upon reasonable request. Data are located in controlled access data storage at CNIO & ICO/Hospital de Bellvitge.
