# Supplementary material for: Denosumab as an immune modulator in HER2-negative early breast cancer: results of the window-of-opportunity D-BIOMARK clinical trial
Source: Breast Cancer Res. 2025 May 12;27:68. doi: 10.1186/s13058-025-01996-w (PMC12067755; doi:10.1186/s13058-025-01996-w)
Supplement: Supplementary file 1 — Additional file 1. [file 13058_2025_1996_MOESM1_ESM.pdf]

Supplementary Data

Table S1. **Adverse events reported during the study. No grade 4 or 5 toxicities were observed.**

Toxicity	Control n=21		Experimental n=37	
	G1-2	G3	G1-2	G3
Hematoma secondary to breast biopsy	2 (9.5%)	0	3 (8.1%)	0
Bone pain	0	0	10 (27.03%)	0
Pain in the site of denosumab infusion	0	0	3 (8.1%)	0
Asthenia	0	0	4 (10.81%)	0
Chill	0	0	2 (5.41%)	0
Tooth infection	0	0	1 (2.70)*	0

* Outside the study follow-up period, at 11 months after denosumab infusion, the same patient presented osteonecrosis of the jaw (ONJ) requiring surgery. This was reported as a possible late adverse event related to denosumab, ONJ was classified as grade 3.

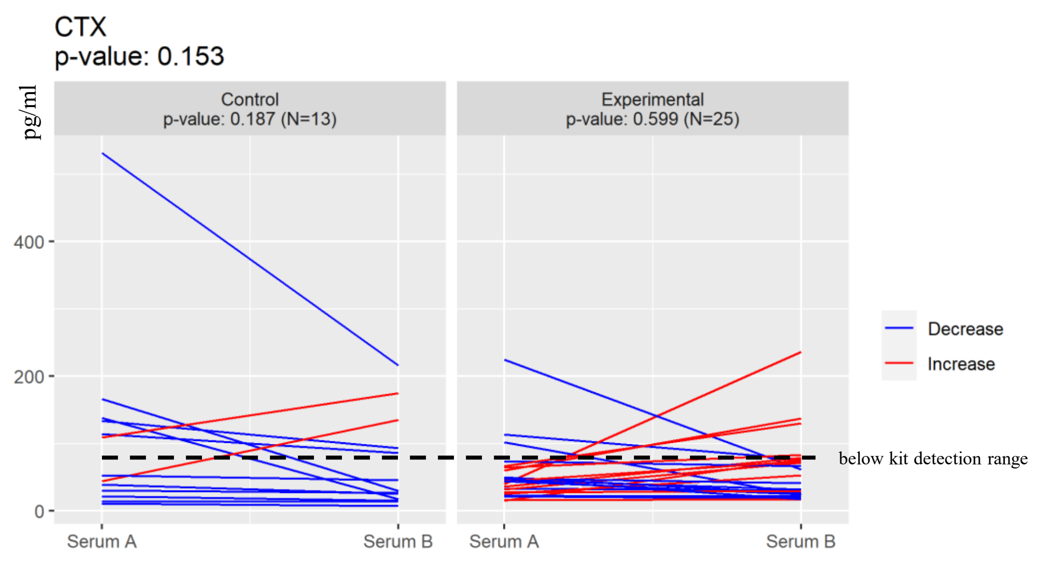

Figure S1**. Serum biomarker carboxy-terminal collagen crosslinks (CTX):** Levels of CTX in serum from patients collected at the time of biopsy (Serum A) and surgery (Serum B) in the control and experimental arms. p value T-test for comparison between experimental and control arm is shown in the upper left corner and p value T-test for paired samples is shown for each treatment group**.** N indicates the number of samples analyzed. **Note that denosumab did not associate with a reduction in the serum levels of CTX, but many baseline samples were already below the detection range of the assay kit.**

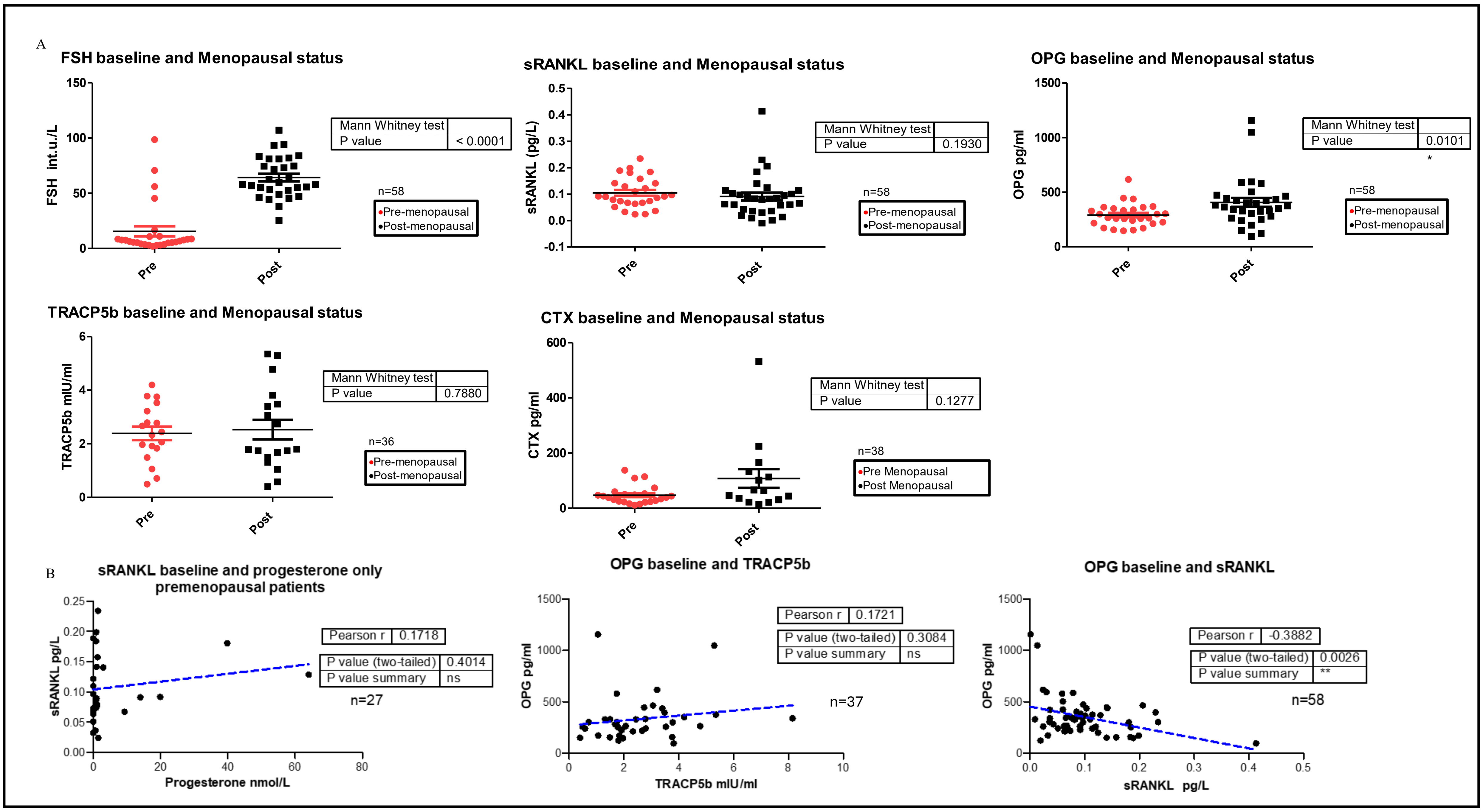

Figure S2. **Baseline levels and correlations of serum follitropin (FSH), sRANKL, OPG, TRACP5b and CTX and menopausal status.** A) Levels of FSH, sRANKL, OPG, TRACP-5b and CTX detected by ELISA, in serum from patients collected at the time of biopsy, attending to the menopausal status. Postmenopausal patients had higher FSH and OPG levels, while sRANKL and TRACP5b levels were comparable between pre- and post-menopausal women. Mann Whitney test p values are shown B) Correlations between levels of indicated markers in serum. Two tailed p values and Pearson r are shown, and n indicates total number of samples. **There were no discernible associations observed between progesterone levels and sRANKL in premenopausal patients, and between OPG and TRACP5b. However, it is noteworthy that sRANKL levels exhibited an inverse relationship with OPG levels.**

Table S2. **Analysis by surrogate molecular subtype.**

	Luminal A-like (N=27)							Luminal B-like (N=21)							TNBC (N=10)						
	Control (N=8)			Experimental (N=19)			p-inter*	Control (N=9)			Experimental (N=12)			p-inter*	Control (N=4)			Experimental (N=6)			p-inter*
	Biopsy	Surgery	p-value	Biopsy	Surgery	p-value		Biopsy	Surgery	p-value	Biopsy	Surgery	p-value		Biopsy	Surgery	p-value	Biopsy	Surgery	p-value	
KI 67 (%)	16.25	21.88	0.125	11.58	14.84	0.126	0.547	25.89	30.33	0.254	30.67	34.92	0.350	0.973	38.00	41.25	0.517	28.67	40.17	0.025	0.197
Cleaved Caspase- 3 (H-score)	1.77	2.55	0.252	1.26	0.96	0.346	0.152	1.19	1.42	0.254	3.79	2.48	0.348	0.275	2.48	3.43	0.350	4.52	4.66	0.694	0.434
sRANKL (pg/L)	0.10	0.11	0.775	0.10	0.00	0.000	0.004	0.08	0.12	0.069	0.10	0.00	0.000	0.000	0.14	0.11	0.282	0.08	0.00	0.028	0.118
OPG (pg/ml)	327.35	320.48	0.556	308.15	311.54	0.690	0.472	384.57	326.29	0.135	301.32	311.78	0.468	0.097	570.52	472.91	0.286	440.48	497.97	0.061	0.129
TRACP5b (mlU/ml)	2.39	2.98	0.342	1.63	2.34	0.102	0.870	3.23	3.48	0.680	2.70	2.58	0.223	0.546	2.75	1.97	0.034	3.40	2.95	0.316	0.437

*T-test for non-paired samples used for comparison between the experimental and control arms.

Table S3. **Analysis by menopausal status only in luminal tumors**

	ER+ Pre-menopausal (N=26)							ER+ Post-menopausal (N=22)						
	Control (N=10)			Experimental (N=16)			p-inter*	Control (N=7)			Experimental (N=15)			p-inter*
	Biopsy	Surgery	p-value	Biopsy	Surgery	p-value		Biopsy	Surgery	p-value	Biopsy	Surgery	p-value	
KI 67 (%)	20.50	29.40	0.031	18.69	22.81	0.244	0.338	22.57	22.00	0.668	19.27	22.40	0.199	0.177
Cleaved Caspase- 3 (H-score)	1.47	2.33	0.109	3.15	1.71	0.169	0.050	1.45	1.42	0.898	1.26	1.38	0.689	0.686
sRANKL (pg/L)	0.09	0.14	0.070	0.11	0.00	0.000	0.000	0.09	0.08	0.803	0.09	0.00	0.000	0.002
OPG (pg/ml)	318.40	293.82	0.107	281.63	288.42	0.561	0.095	413.70	366.02	0.337	330.98	336.38	0.573	0.295
TRACP5b (mlU/ml)	3.09	3.64	0.256	2.18	2.23	0.677	0.312	2.47	2.59	0.889	2.15	2.76	0.239	0.628

*T-test for non-paired samples used for comparison between the experimental and control arms.

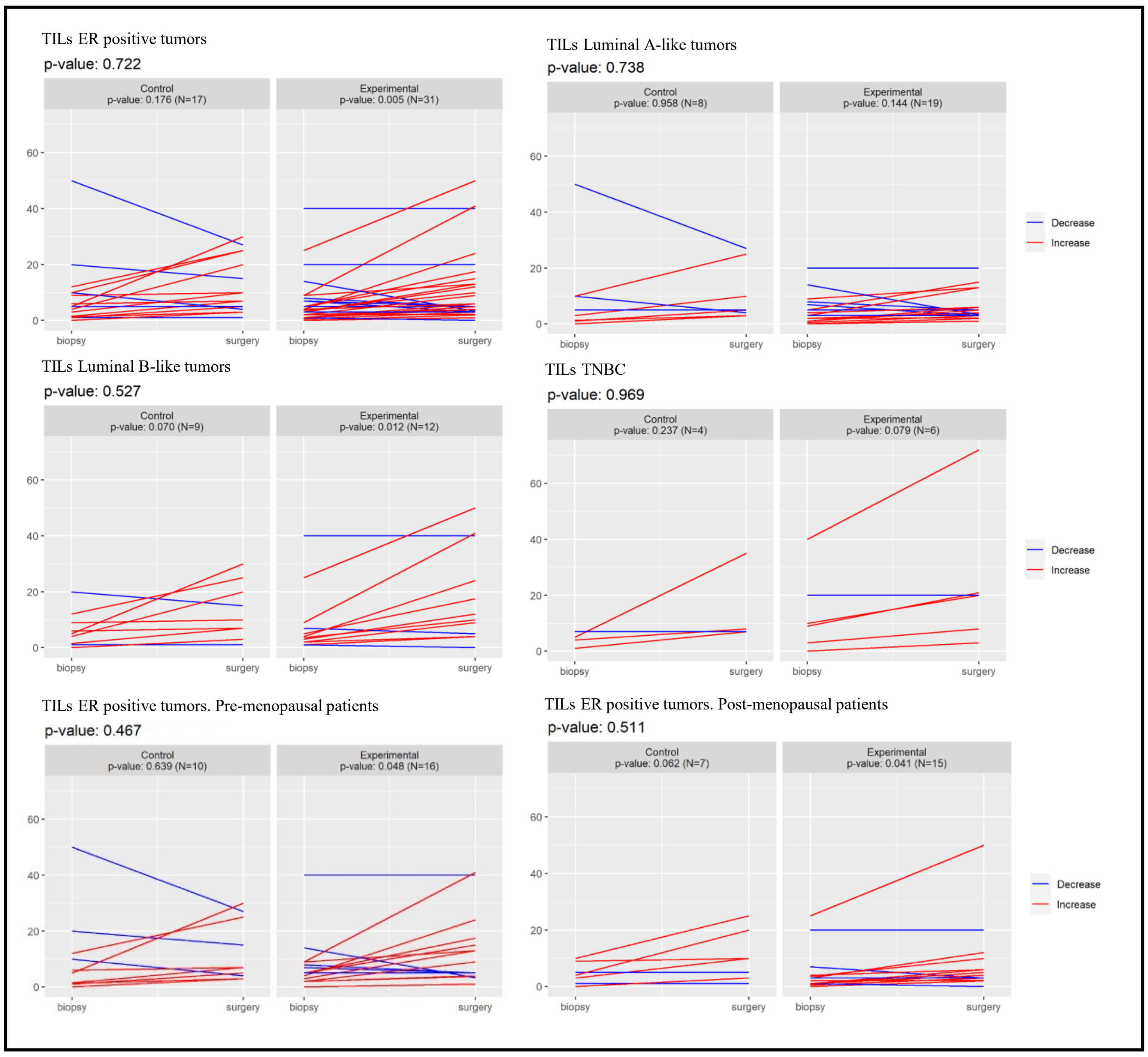

Figure S3. **Analysis of tumor-infiltrating lymphocytes (TILs) by surrogate molecular subtype and menopausal status**. The data show that, in all cases except for triple-negative (TNBC) and luminal A-like tumors, the experimental group exhibits a statistically significant increase in TILs. p value T-test for comparison between experimental and treatment arm is shown in the upper left corner and p value T-test for paired samples is shown for each treatment group**.** N indicates the number of samples analyzed. **Note that only the experimental group exhibited a statistically significant increase in the percentage of TILs.**

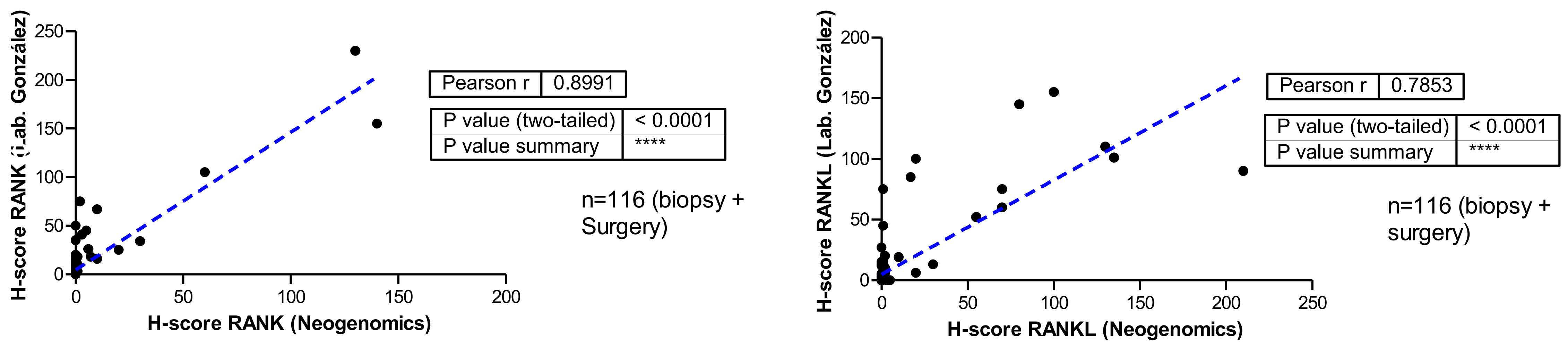

Figure S4. **Correlations between quantifications of RANK and RANKL expression in tumor cells (H-score).** Quantifications were performed by Negenomics Laboratory (outsourced) and by Dr. González-Suárez's Laboratory. Pearson r and two tailed p value are indicated. Similar results observed in both quantifications.

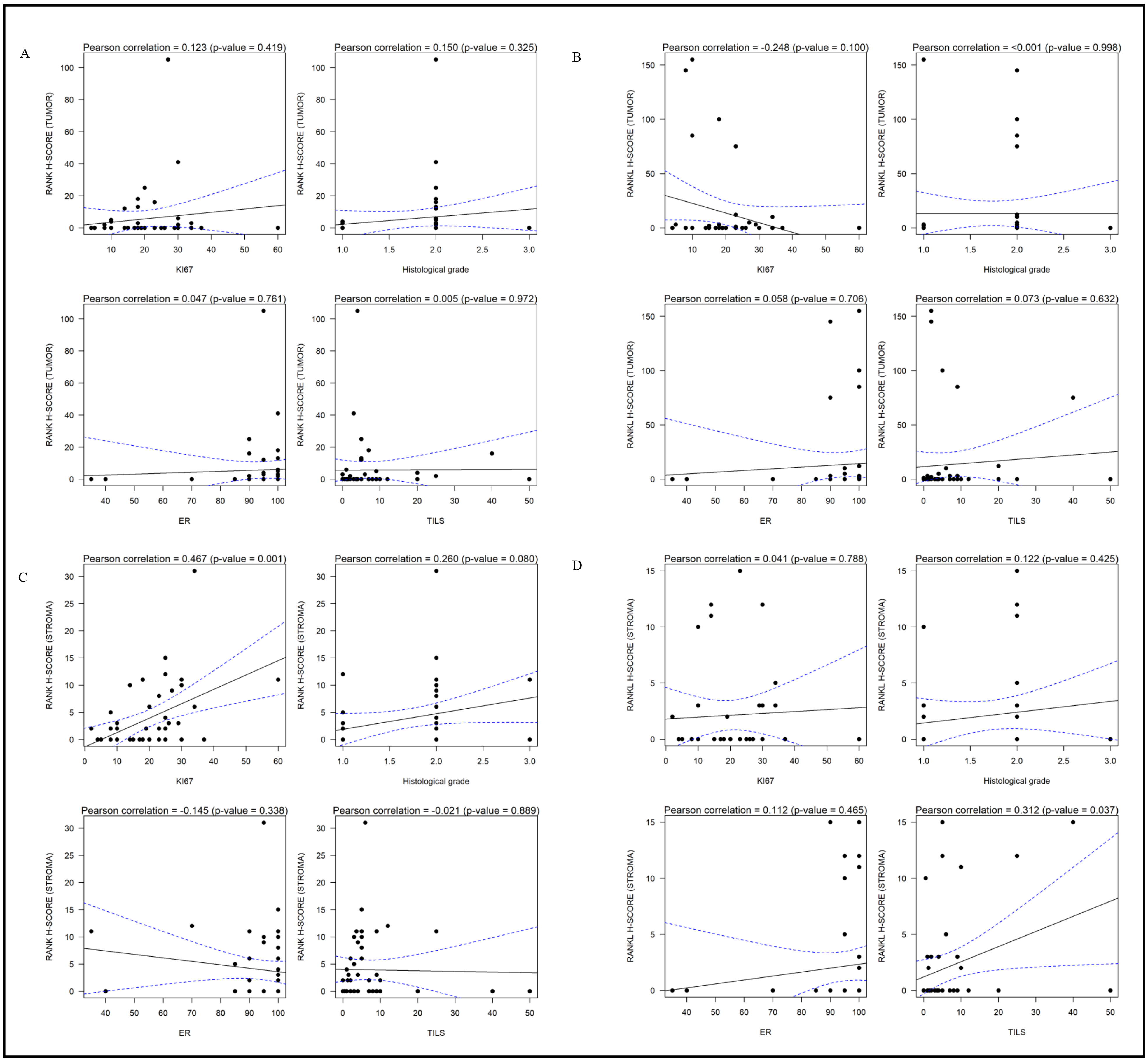

Figure S5. **Correlation analysis between RANK/RANKL expression in tumor (A,B) or stroma (C,D) and clinicopathological parameters exclusively in luminal tumors.** A and B: Correlation analysis between RANK/RANKL expression in tumor with Ki67, histological grade, ER and TILs using Pearson's correlation coefficient. C and D: Correlation analysis between RANK/RANKL expression in stroma with Ki67, histological grade, ER and TILs using Pearson's correlation coefficient. **Notably, RANK expression in stromal cells of ER-positive breast samples positively correlated with Ki67. Additionally, RANKL expression in the stroma was associated with TILs.**

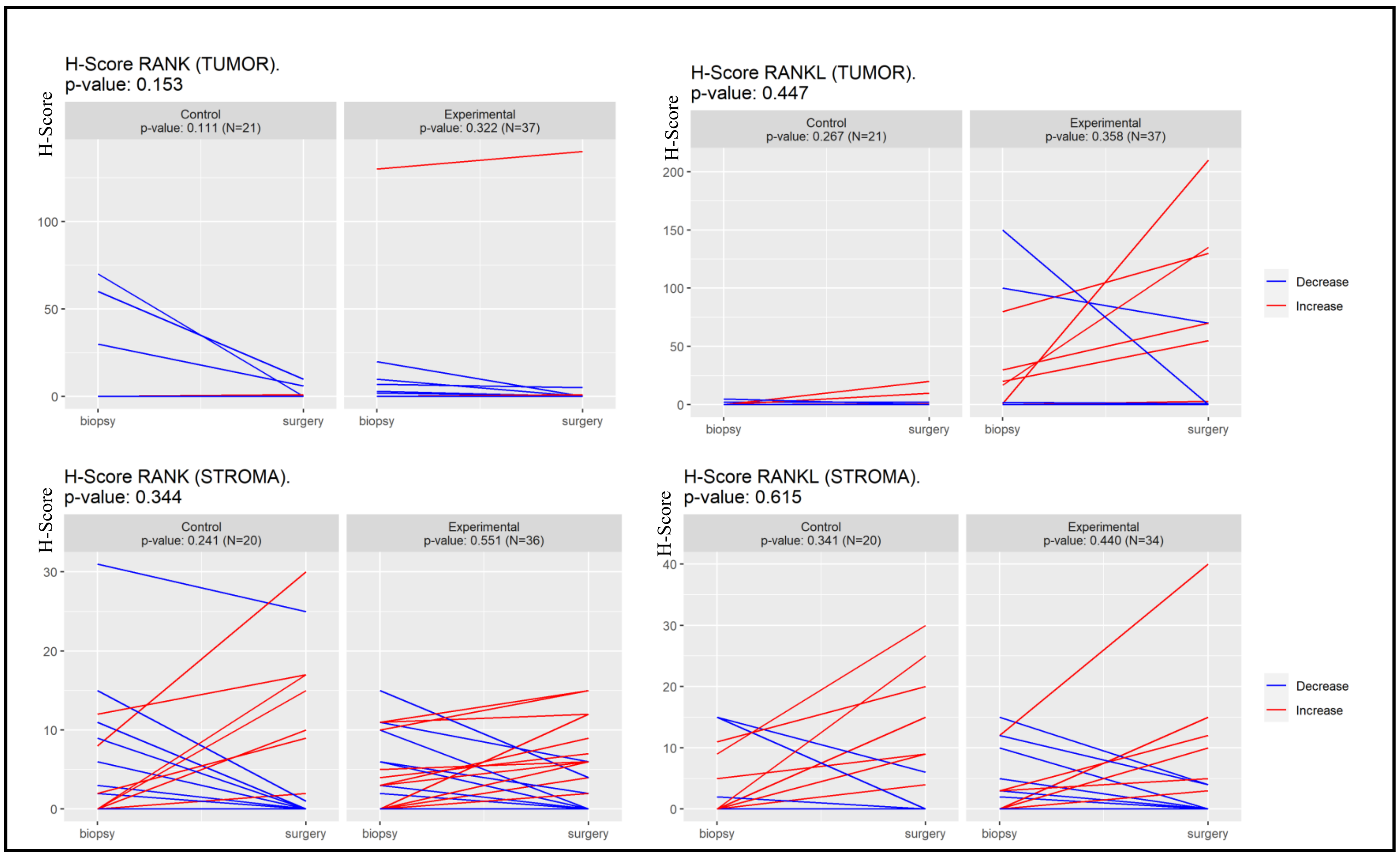

Figure S6: **RANK and RANKL expression (H–scores) in tumor and stroma.** RANK and RANKL expression (H-Score) detected by IHC in tumor cells and stroma at the diagnostic biopsy (biopsy) and in the surgical specimen (surgery) in the control and experimental arm. p value T-test for comparison between experimental and treatment arm is shown in the upper left corner and p value T-test for paired samples is shown for each treatment group**.** N indicates the number of samples analyzed**. No significant changes in the expression of RANK and RANKL were observed between biopsy and surgery.**
